# Anatomical Analysis of Transient Potential Vanilloid Receptor 1 (*Trpv1*+) and Mu-Opioid Receptor (*Oprm1*+) Co-expression in Rat Dorsal Root Ganglion Neurons

**DOI:** 10.3389/fnmol.2022.926596

**Published:** 2022-07-07

**Authors:** Wenting Ma, Matthew R. Sapio, Allison P. Manalo, Dragan Maric, Mary Kate Dougherty, Taichi Goto, Andrew J. Mannes, Michael J. Iadarola

**Affiliations:** ^1^Department of Perioperative Medicine, Clinical Center, National Institutes of Health, Bethesda, MD, United States; ^2^National Institute of Neurological Disorders and Stroke, Flow and Imaging Cytometry Core Facility, Bethesda, MD, United States; ^3^Symptoms Biology Unit, National Institute of Nursing Research, National Institutes of Health, Bethesda, MD, United States

**Keywords:** dorsal root ganglia (DRG), opioid receptor, transient receptor potential vanilloid-1 (TRPV1), nociception, transient receptor potential A1 (TRPA1), mu opioid (MOP) receptor, Kappa opioid receptor (KOR), TRPM8

## Abstract

Primary afferent neurons of the dorsal root ganglia (DRG) transduce peripheral nociceptive signals and transmit them to the spinal cord. These neurons also mediate analgesic control of the nociceptive inputs, particularly through the μ-opioid receptor (encoded by *Oprm1*). While opioid receptors are found throughout the neuraxis and in the spinal cord tissue itself, intrathecal administration of μ-opioid agonists also acts directly on nociceptive nerve terminals in the dorsal spinal cord resulting in marked analgesia. Additionally, selective chemoaxotomy of cells expressing the TRPV1 channel, a nonselective calcium-permeable ion channel that transduces thermal and inflammatory pain, yields profound pain relief in rats, canines, and humans. However, the relationship between *Oprm1* and *Trpv1* expressing DRG neurons has not been precisely determined. The present study examines rat DRG neurons using high resolution multiplex fluorescent *in situ* hybridization to visualize molecular co-expression. Neurons positive for *Trpv1* exhibited varying levels of expression for *Trpv1* and co-expression of other excitatory and inhibitory ion channels or receptors. A subpopulation of densely labeled *Trpv1*+ neurons did not co-express *Oprm1*. In contrast, a population of less densely labeled *Trpv1*+ neurons did co-express *Oprm1*. This finding suggests that the medium/low *Trpv1* expressing neurons represent a specific set of DRG neurons subserving the opponent processes of both transducing and inhibiting nociceptive inputs. Additionally, the medium/low *Trpv1* expressing neurons co-expressed other markers implicated in pathological pain states, such as *Trpa1* and *Trpm8*, which are involved in chemical nociception and cold allodynia, respectively, as well as *Scn11a*, whose mutations are implicated in familial episodic pain. Conversely, none of the *Trpv1*+ neurons co-expressed *Spp1*, which codes for osteopontin, a marker for large diameter proprioceptive neurons, validating that nociception and proprioception are governed by separate neuronal populations. Our findings support the hypothesis that the population of *Trpv1* and *Oprm1* coexpressing neurons may explain the remarkable efficacy of opioid drugs administered at the level of the DRG-spinal synapse, and that this subpopulation of *Trpv1*+ neurons is responsible for registering tissue damage.

## Introduction

The nociceptive system is a major target of interest for the design of analgesic drugs. When administered at this level of the pain pathway, analgesic agents such as opioids and local anesthetics ultimately act on primary afferent endings by inhibiting presynaptic release of nociceptive signaling molecules, such as substance P and calcitonin gene-related peptide (CGRP) (Julius and Basbaum, 2001; Kondo et al., 2005). A better understanding of which afferents are required for nociception and what constellation of receptors are expressed by these neurons is an important component of designing alternative strategies to selectively inhibit nociception while preserving other somatosensory functions.

Transient receptor potential vanilloid subfamily, member 1 (TRPV1) is expressed by primary afferent nociceptive neurons (Caterina et al., 1997; Szallasi and Blumberg, 1999; Caterina, 2007), and transduces thermal and inflammatory tissue damage stimuli (Karai et al., 2004a; Mishra and Hoon, 2010; Mitchell et al., 2010, 2014). In the search for novel non-opioid analgesics, much attention has been centered on the TRPV1 molecule as an analgesic target (Jancso et al., 1977; Yaksh et al., 1979; Bevan et al., 1992; Urban et al., 2000; Wong and Gavva, 2009; Iadarola and Gonnella, 2013). Genetic ablation or chemical silencing of *TRPV1*+ afferents has been reported to cause loss of nociceptive responses (Jancso et al., 1977; Karai et al., 2004a; Brown et al., 2005, 2015; Mishra and Hoon, 2010; Brown and Iadarola, 2015; Sapio et al., 2018). However, direct antagonism of the TRPV1 receptor has not led to clinically useful antinociceptive agents, despite promising preclinical rodent studies and demonstrable target engagement in humans (Menéndez et al., 2006; Szallasi et al., 2007; Rowbotham et al., 2011; Quiding et al., 2013). These drugs did not advance in part due to induction of varying degrees of hyperthermia (Wong and Gavva, 2009; Garami et al., 2018; Park et al., 2021), and lack of sustained efficacy (Quiding et al., 2013), as TRPV1 is not the only nociceptive transducing molecule expressed by primary afferents (Goswami et al., 2014). In the complex milieu of tissue damage and inflammation, multiple other algesic mediators remain capable of discharging the primary afferents, even with a high degree of TRPV1 inhibition, which may explain why TRPV1 antagonists are much less analgesic than agonist-mediated chemoaxotomy approaches (Karai et al., 2004a; Iadarola et al., 2018; Sapio et al., 2018). Additionally, patients treated with high concentrations of TRPV1 antagonists become insensitive to noxious hot thermal stimuli, posing a burn risk as they rated temperatures up to 49°C as safe (Rowbotham et al., 2011). Thus, TRPV1+ afferents are the critical transducers of pain associated with tissue damage as well as most forms of warm and hot thermosensation.

Untangling desirable and undesirable pharmacologic effects of TRPV1-based therapies requires clarification of the underlying neurobiology. An interesting observation is that selective lesioning of TRPV1+ nerve endings using the highly potent TRPV1 agonist, resiniferatoxin (RTX), leads to loss of thermal nociceptive sensitivity and with high subcutaneous doses (250 ng intraplantar) can produce near-total insensitivity to tissue-damaging stimuli in the denervated area (Mitchell et al., 2010, 2014). While it is known that TRPV1+ nociceptors comprise several discrete populations of sensory afferent neurons (Sapio et al., 2018), detailed studies of nociceptive cellular neurobiology are needed to elucidate the precise molecular markers of each population. The objective of the present investigations is to delineate the molecular complement of receptors and ion channels in neurons responsible for nociception and analgesic action. Specifically, we hypothesize that we can identify a population of cells using multiplex labeling that contains several nociceptive and analgesic markers, that maybe useful for future studies of pain and analgesia. Delineating these neurons in a quantitative, molecularly-defined fashion enables future studies to directly target these neurons using selective pharmacological analgesic strategies.

## Materials and Methods

### Rat Dorsal Root Ganglia Tissue Procurement and Slide Preparation

Experiments in the present study were approved by the Institutional Animal Care and Use Committee of the Clinical Center, National Institutes of Health (Bethesda, Maryland). Male Sprague-Dawley rats 7–8 weeks old weighing 175–200 g (Charles River Laboratories, Wilmington, MA) were used for all experiments. Rats were housed in pairs with a plastic tube for enrichment/housing. At the time of tissue collection, rats were euthanized under deep isoflurane anesthesia (≥4%) and perfused with cold intracardiac PBS followed by 10% formalin. Bilateral lumbar DRGs were harvested at the level of L3–L6. DRGs were fixed in 10% formalin for 24 h and subsequently transferred to 2.5% formalin before embedding (within 3–4 days). DRG tissues were embedded in paraffin blocks and cut to 6 μm sections and mounted on slides by Histoserv, Inc. (Germantown, MD). For these experiments DRGs from *N* = 6 rats were collected; with *N* = 3 stained, analyzed, and scanned for most analyses. Cell types identified were found in approximately the same proportions in each individual animal. Multiple windows on the sections of DRG were selected to capture representative fields for quantification. Sample sizes were estimated from previous experiments (Sapio et al., 2020b).

### Fluorescent Multiplex *in situ* Hybridization and Microscopic Imaging

Fluorescent RNA *in situ* hybridization was performed using RNAScope^®^ Multiplex Fluorescent V2 Assay (Advanced Cell Diagnostics, Newark, CA) in accordance with the manufacturer's instructions for treating formalin-fixed paraffin-embedded tissue. Target retrieval was performed for 15 mins at 100°C. The catalog numbers of the probes used in these experiments are listed in Table 1. Note that color balancing was performed based on sFPKM data (Sapio et al., 2016) to put weaker signals in channels with the least background autofluorescence (higher wavelengths) to avoid signal bleed. To avoid cross reaction between opioid receptors, the kappa opioid receptor (*Oprk1*) probe was custom designed to be specific when used together with the other opioid receptor detection probes. This was accomplished by targeting base pairs 1,300–2,293 of NM_017167.3. To validate a neuronal population exhibiting a low signal for *Trpm8* hybridization, a second probe was used against a different region of the transcript. This was targeted to base pairs 2,671–3,590 of NM_134371.3.

**Table 1:** Table of genes examined in the present study.

**Probes used for** ***in situ*** **hybridization**			
**Gene name**	**Symbol**	**Catalog#**	**sFPKM**
Delta Opioid Receptor 1	*Oprd1*	457011-C3	1.5
Kappa Opioid Receptor 1	*Oprk1*	1037891-C1	3.2
Opioid Related Nociceptin Receptor 1	*Oprl1*	576011	25.8
Mu Opioid Receptor 1	*Oprm1*	410691-C2	7.9
Sodium Voltage-Gated Channel Alpha Subunit 11	*Scn11a*	404811-C2	121.5
Secreted Phosphoprotein 1	*Spp1*	405441-C2	1179.0
Transient Receptor Potential Cation Channel, Subfamily A (Ankyrin-like) Member 1	*Trpa1*	312511-C2	23.0
Transient Receptor Potential Cation Channel Subfamily M (Melastatin) Member 8	*Trpm8*	539571 and 1127541	51.4
Transient Receptor Potential Cation Channel Subfamily V (Vanilloid) Member 1	*Trpv1*	501161-C4	58

*Expression data in significant fragments per kilobase per million aligned reads (sFPKMs) comes from a previously published study (Sapio et al., 2016). This expression data was used at the experimental design stage for color balancing. Note that two Trpm8 probes were used to validate the low intensity staining result from the Trpm8 stain*.

Hybridized slides were imaged using an Axio Imager.Z2 scanning fluorescence microscope (Zeiss, Oberkochen, Germany), fitted with an ORCA-Flash4.0s CMOS sensor high resolution digital camera (Hamamatsu, Shizuoka, Japan), 20X/0.8 Plan-Apochromat (Phase-2) non-immersion objective (Zeiss), and 200W X-Cite 200DC broad band lamp source (Excelitas Technologies, Waltham MA). Filter sets (Semrock, Rochester NY) for detecting DAPI, Opal520, Opal570, Opal620, and Opal690 fluorescent dyes (Opal Reagent Systems; PerkinElmer, Waltham MA) were custom furnished as described previously (Maric et al., 2021) (Supplementary Table 1). Image tiles sized 600 × 600 μm were captured and seamlessly stitched using ZEN2 imaging software (Zeiss) at 3 pixel/μm spatial resolution. For each emission wavelength, fluorescent microscopy images were captured and displayed as individual layers to generate multi-colored composites. Representative images are enhanced for visibility.

### Quantification and Visualization of *in situ* Data

The images were processed with Adobe Photoshop and Fiji (Image J v2.1.0/1.53c) in order to analyze the co-localization patterns of genes that are detected in the multiplex *in situ* hybridization experiments. For quantification of signal intensity inside individual DRG neurons, polygons were drawn based on the bright field image in a multichannel overlay using Fiji, and fluorescence intensity of this polygonal area was measured. The neuronal diameter was extrapolated from the polygonal area using the formula for the diameter of a circle [diameter = 2√ (area/π)]. For cell counting, cells were counted manually in Photoshop, and tabulated counts were used to plot visualizations of intersecting sets in R (v4.05) using the UpSet package. In these plots, the number of cells positive for each of the four markers is plotted as “set size” and the number of cells co-positive for each given combination of markers is shown as “intersection size.” Cells were identified using a combination of DAPI-labeling and diffusion image contrast imaging which revealed cellular outline. A single 6 μm section was counted for each stain per rat to avoid counting the same cell twice in serial sections. Statistical testing of diameter measurements was conducted using Prism GraphPad (Version 9.3.0, Kruskal-Wallis test with Dunn's multiple comparison test). In these experiments, data from each individual rat was examined before pooling to ensure homogeneity, and the individual values are shown in Supplementary Figure 1. Proportions of cells positive for *Oprm1, Oprk1, Oprd1*, and *Trpv1* markers were determined in a pairwise manner and represented as percentages relative to the total number of cells counted.

To distinguish cell populations by levels of *Trpv1, Oprm1, Oprd1*, and *Trpm8* expression, a surface plot was generated in Fiji to visualize fluorescence intensity per pixel. Surface plots have three axes, with the x and y axes corresponding to the physical dimension of the slide section, and the z-dimension, or height of the surface representing intensity. Intensity in these plots is also represented by color in a flame scale.

For quantitative graphs, each channel was checked for non-specific signal coming from neighboring channels and subtracted if signal bleed was detected to quantify only specific fluorescent signal.

## Results

### Co-expression of Transient Receptor Potential Vanilloid 1 (*Trpv1*) and the Three Opioid Receptors (*Oprm1, Oprk1, Oprd1*)

**Figure 1:**
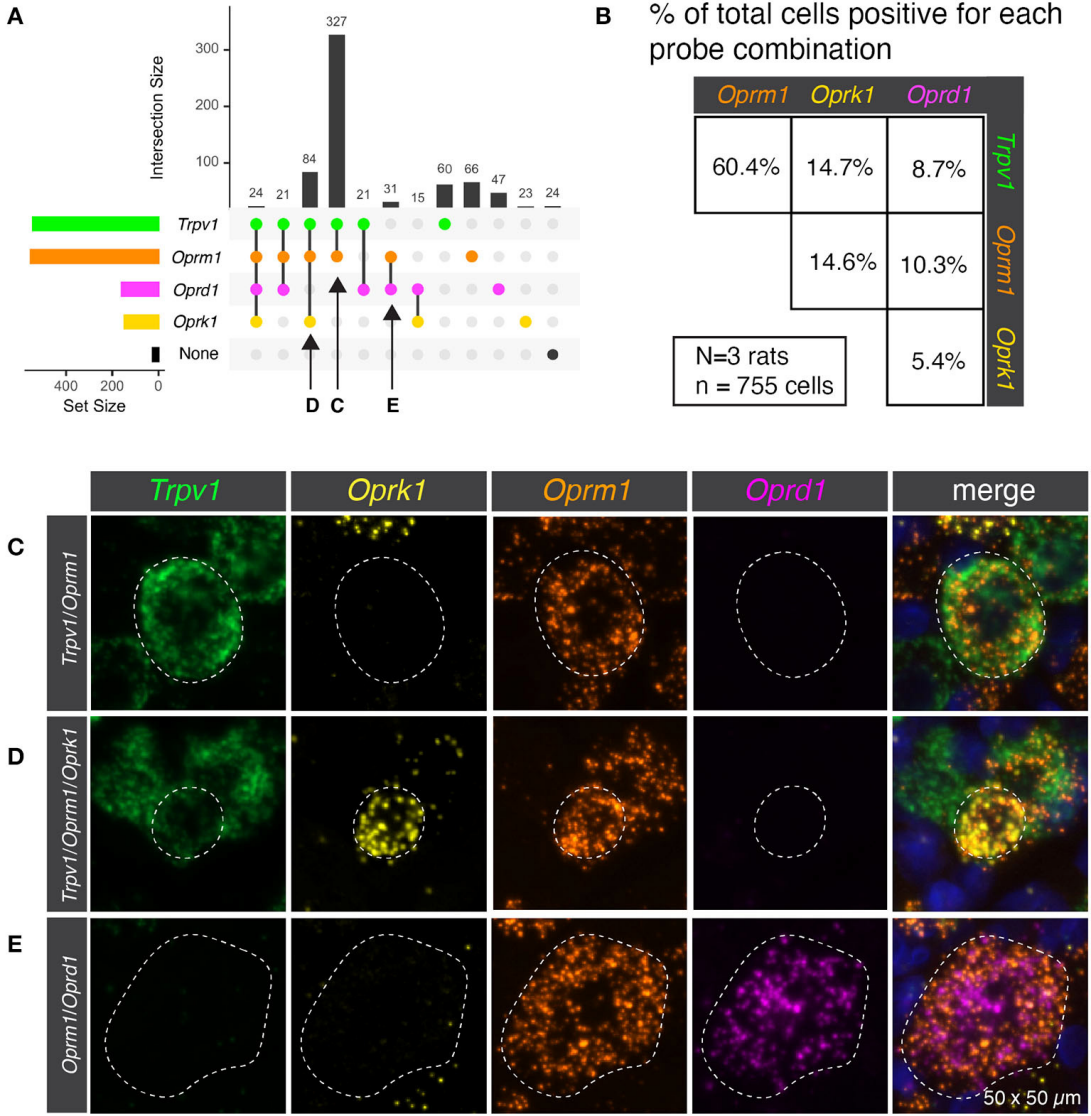
Co-expression of transient receptor potential vanilloid 1 (*Trpv1*) and the three opioid receptors (*Oprm1, Oprk1, Oprd1*). **(A)** Semiquantitative analysis was performed to count the number of cells present or absent for each of the four marker genes. This analysis subdivided the sensory afferents into 12 populations, although not all of these populations were prevalent. **(B)** In order to simplify the overlap between *Trpv1* and Opioid receptor transcripts, a pairwise matrix of percentages is shown. This shows the total number of neurons positive for each marker (set size) as well as the number of cells positive for combinations of markers (intersection size; see Methods section). This display clarifies that *Trpv1*+/*Oprm1*+ cells were very prevalent in the DRG, comprising 60.4% of all neurons (ignoring categorization by the other markers.). While all possible combinations were identified, some were quite rare, with the population of cells positive for both *Oprk1* and *Oprd1* (the rarest combination) comprising ~5% of neurons. **(C)** Representative multi-channel microscopy images are shown for three of the populations identified in panel A, the first of which being *Trpv1*+*/Oprm1*+ neurons. These neurons were the most abundant population at 327 of 755 total cells (or 43.3%). **(D)** The *Trpv1*+*/Oprm1*+/*Oprk1*+ population is shown, which is the second most abundant subpopulation at 84/755 cells (11.1%). **(E)** Finally, a representative cell from the *Oprm1*+*/Oprd1*+ population is shown. This population is rare, but is large and expresses very high levels of both *Oprm1* and *Oprd1*. Outlines of neurons are shown to enhance visibility (dotted lines).

Semiquantitative analysis was performed to estimate the number of cells present or absent for the mRNA encoding the thermosensory nociceptive ion channel *Trpv1* and each of the three opioid receptors (*Oprm1, Oprk1*, and *Oprd1*). This analysis subdivided the sensory afferents into 12 populations, though not all were prevalent (Figure 1A). In order to concisely depict the overlap between *Trpv1* and opioid receptor transcripts, a pairwise matrix of percentages was constructed (Figure 1B). The most prevalent binary combination of markers was *Trpv1* and *Oprm1*, comprising 60.4% of all neurons (ignoring categorization by the other markers). The κ-opioid receptor (encoded by *Oprk1*) was identified with ~15% of neurons in combination with either *Trpv1* or *Oprm1*. The δ-opioid receptor (*Oprd1*) was less prevalent, and the rarest combination were neurons co-positive for δ- and κ-opioid receptors (5.4%). These results are based on the aggregated quantification of 755 cells from *N* = 3 rats. The bottom 15 panels show representative images of the distinct co-expressing populations of *Trpv1*+*/Oprm1*+ neurons (Figure 1C), *Trpv1*+*/Oprm1*+*/Oprk1*+ neurons (Figure 1D), and *Oprm1*+*/Oprd1*+ neurons (Figure 1E).

### Heterogeneous Levels of *Trpv1* mRNA in Rat DRG

**Figure 2:**
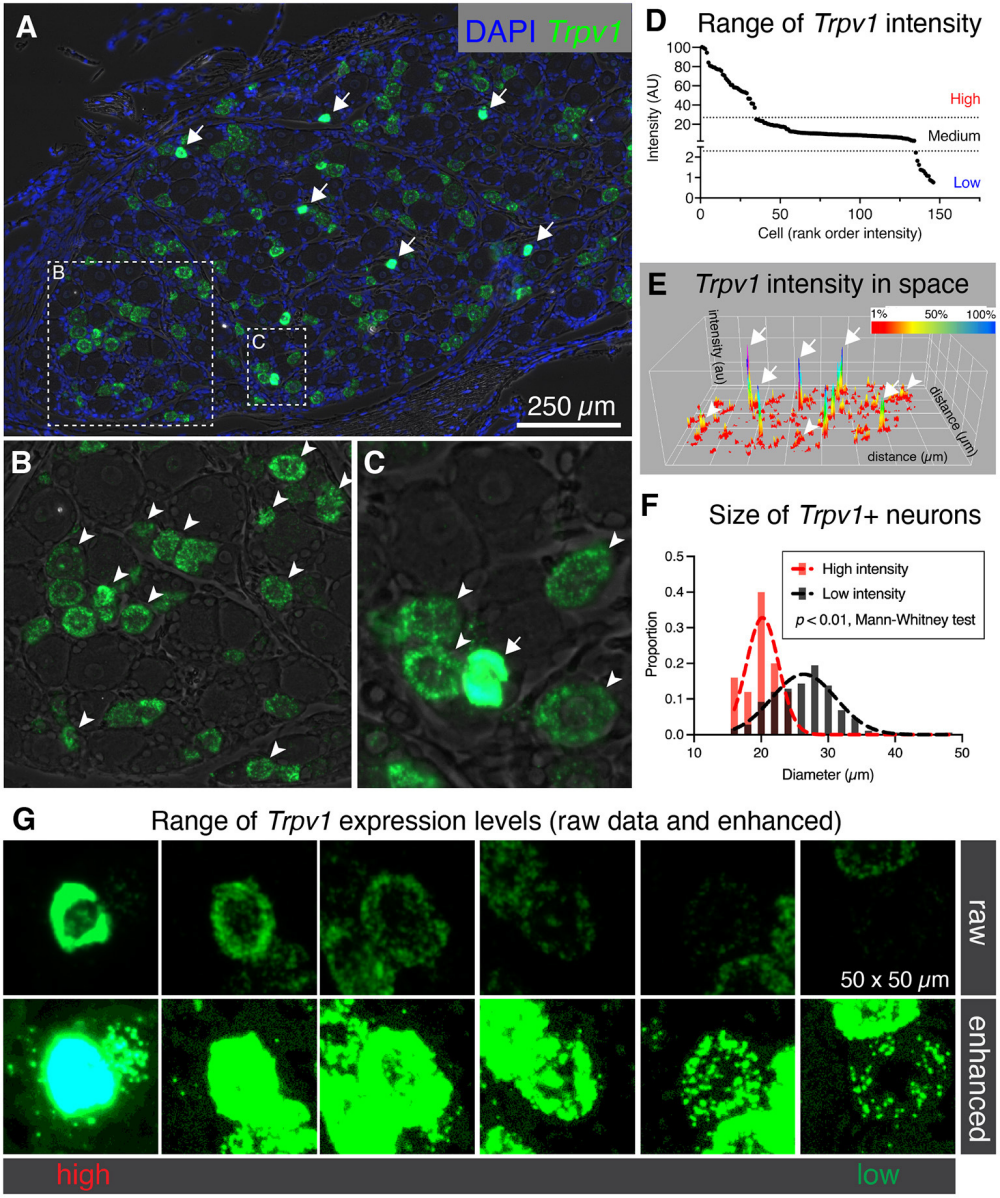
Heterogeneous labeling of *Trpv1* mRNA in rat dorsal root ganglion (DRG). **(A)** Fluorescent *in situ* hybridization was performed and imaged for whole rat DRG sections (*N* = 3). Very high levels of expression were observed in a small number of small diameter neurons (white arrows). **(B)** An enlargement of a neuron-rich region is shown with *Trpv1*+ neurons (arrowheads). **(C)** A second enlargement includes one high-expressing *Trpv1*+ neuron (arrow) and several other Trpv1+ neurons with moderate staining intensity (arrowheads). **(D)** Intensity values for *Trpv1* were plotted by rank order. Based on these values, *Trpv1* expression was divided into high, medium and low expression. **(E)** A surface plot was generated for *Trpv1* intensity (arbitrary units, Z-axis) across the entire DRG section shown in **(A)**. This plot shows the relationship between the high-expressing *Trpv1* DRG neurons and other cells in the ganglion. Note the high peaks indicating a quantitatively separate population (white arrows). **(F)** Diameter of the high intensity *Trpv1*+ neurons was examined by measuring the area in Fiji. High intensity *Trpv1*+ neurons had a stereotyped small diameter as represented by the narrow Gaussian (red) relative to the broader distribution of medium/low *Trpv1*+ neurons (black Gaussian). This difference was significant based on a Mann-Whitney test (*p* < 0.01). **(G)** Representative fields of 6 *Trpv1*+ DRG neurons are shown, spanning a range of expression levels. Scanning parameters are tuned so as not to saturate the brightest cells. Note that the range of expression values is such that when the lower expressing *Trpv1*+ neurons are visible, the highest cells are saturated.

While examining binary expression patterns (presence vs. absence), we noted substantial variation in expression of *Trpv1* transcript. Previously, it has been reported that *Trpv1* expression level is high in small diameter thermosensitive C-fibers, consistent with our observation that the highest intensity signals were observed within sparsely distributed small diameter neurons (Figure 2A, arrows). Based on this variation in *Trpv1* expression levels we quantified the fluorescence intensity for *Trpv1* to further characterize if quantitative expression levels of this ion channel gene indicated cell type differences. A lower intensity of *Trpv1* fluorescent signal was evident in many other cells (Figures 2B,C, arrowheads), consistent with the observation of multiple *Trpv1*+ neuronal populations containing a range of expression (Figure 2D) (Cavanaugh et al., 2011a; Goswami et al., 2014; Mitchell et al., 2014). Quantification of fluorescence intensity revealed three loosely separated populations (inflection points or “knees,” in the plot of Figure 2D; *N* = 3 rats, *n* = 146 cells) corresponding to high, medium, and low expressing *Trpv1*+ neurons, consistent with previous findings from single-cell RNA-Seq (Usoskin et al., 2015; Li et al., 2016; Sapio et al., 2018). A surface plot was generated to visualize the range of expression profiles in the DRG neurons. Note that the high expressors appear as isolated peaks in this view, indicating that they are quantitatively separate from other *Trpv1*+ neurons (Figure 2E, arrows). Additionally, high intensity *Trpv1*+ neurons were smaller in diameter than the lower intensity *Trpv1*+ neurons (Figure 2F). In imaging *Trpv1*+ neurons, the exposure was set to minimize oversaturation of the signal at the highest level of expression, leading to apparent low signal in some cells. This is visualized showing the raw data (Figure 2G, upper row) and an enhanced image in which brightness is adjusted (Figure 2G, lower row), which shows the wide dynamic range of the signal. Note that even lower expressing *Trpv1*+ neurons contain a high enough density of puncta to distinguish their signal from background.

### Analysis of Distribution of Intensity Values for the Opioid Receptor Genes *Oprm1* and *Oprd1*

**Figure 3:**
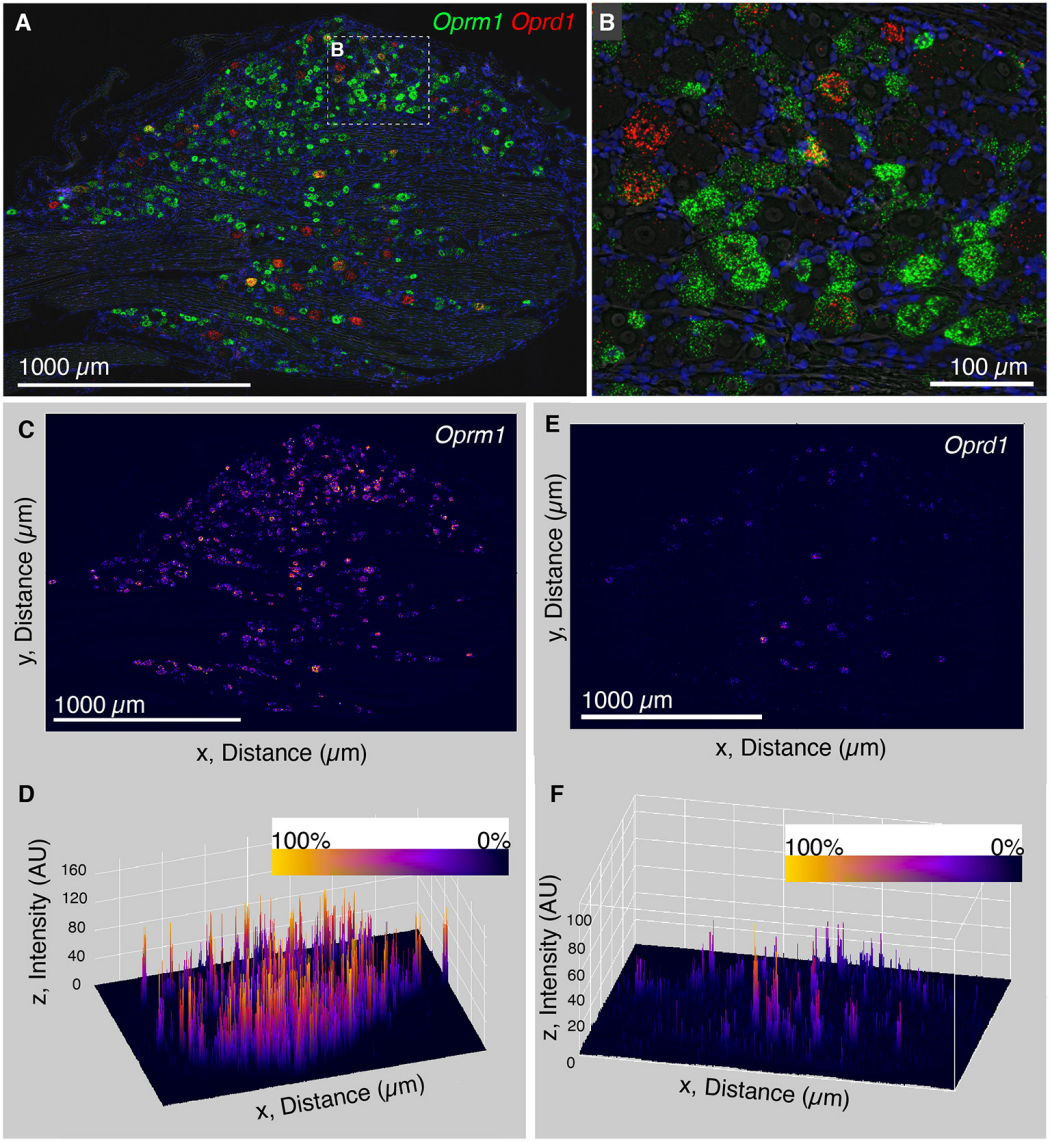
Analysis of intensity distribution for the opioid receptor genes *Oprm1* and *Oprd1*. The finding that *Trpv1* was unevenly distributed in rat DRG prompted an investigation of *Oprm1* and *Oprd1* to answer whether these analgesic opioid receptors were also distributed unevenly across cell populations. **(A)** In a wide field view of a DRG stained for *Oprm1* and *Oprd1*, we did not observe marked differences in expression across the field. The gene encoding the mu-opioid receptor (*Oprm1*) is much more widely expressed than the delta receptor gene (*Opd1*), and both show a range of expression values. **(B)** However, this range appeared qualitative smaller than that observed for *Trpv1*. **(C)** In order to quantify this further, we used a surface plot to represent intensity over area for *Oprm1*. **(D)** When viewed from an angle, this analysis shows many peaks of similar height across the DRG, consistent with the wide expression pattern of *Oprm1*. This is in contrast to *Trpv1*, which showed several very high peaks (Figure 2E). **(E)** The same analysis was also performed for *Oprd1*, which is expressed in many fewer cells than either *Trpv1* or *Oprm1*. **(F)** Viewed at an angle, most of these *Oprd1* peaks are evident in the same linear axis. In Supplementary Figure 2, we quantified differences in distribution and coefficient of variation in these measurements, showing that *Trpv1* had significantly higher coefficient of variation than either *Oprm1* or *Oprd1*.

The finding that *Trpv1* was unevenly distributed in rat DRG prompted an investigation of *Oprm1* and *Oprd1*. We also examined the distribution of expression intensity for two of the analgesic opioid receptors, the μ-opioid receptor transcript (*Oprm1*), and the delta opioid receptor transcript (*Oprd1*), which are responsible for opioid analgesic responses *in vivo* (reviewed in Valentino and Volkow, 2018). In a wide field we show an enhanced zoomed out image (brightened for clarity; Figures 3A,B). From this zoomed out field we did not observe a similar evident difference in expression level analogous to what was observed for *Trpv1* in either *Oprm1* or *Oprd1*. Note that *Oprm1* is expressed in a much greater number of neurons than *Oprd1*. In order to quantify this further, we used a surface plot to represent intensity over area for *Oprm1* (Figures 3C,D) and *Oprd1* (Figures 3E,F). In the angled view, the peaks represent intensity for staining with these labels, and generally fit the overall expression pattern of these receptors with evident peaks corresponding to the majority of positive cells. This is in contrast to what was observed for *Trpv1*, which had a small number of highly evident peaks on a linear axis (Figure 2E). Rank order plots were created to quantify expression, and test whether coefficient of variation differed between measured intensity values for *Trpv1, Oprm1* and *Oprd1*. These analyses showed a greater coefficient of variation in Trpv1 intensity as compared with *Oprm1* or *Oprd1* (Supplementary Figure 2).

### Co-labeling of the Nociceptive Ion Channel Genes *Trpv1* and *Trpa1* With the *Oprm1* Opioid Receptor and the Opioid Receptor-Like *GPCR Oprl1*

**Figure 4:**
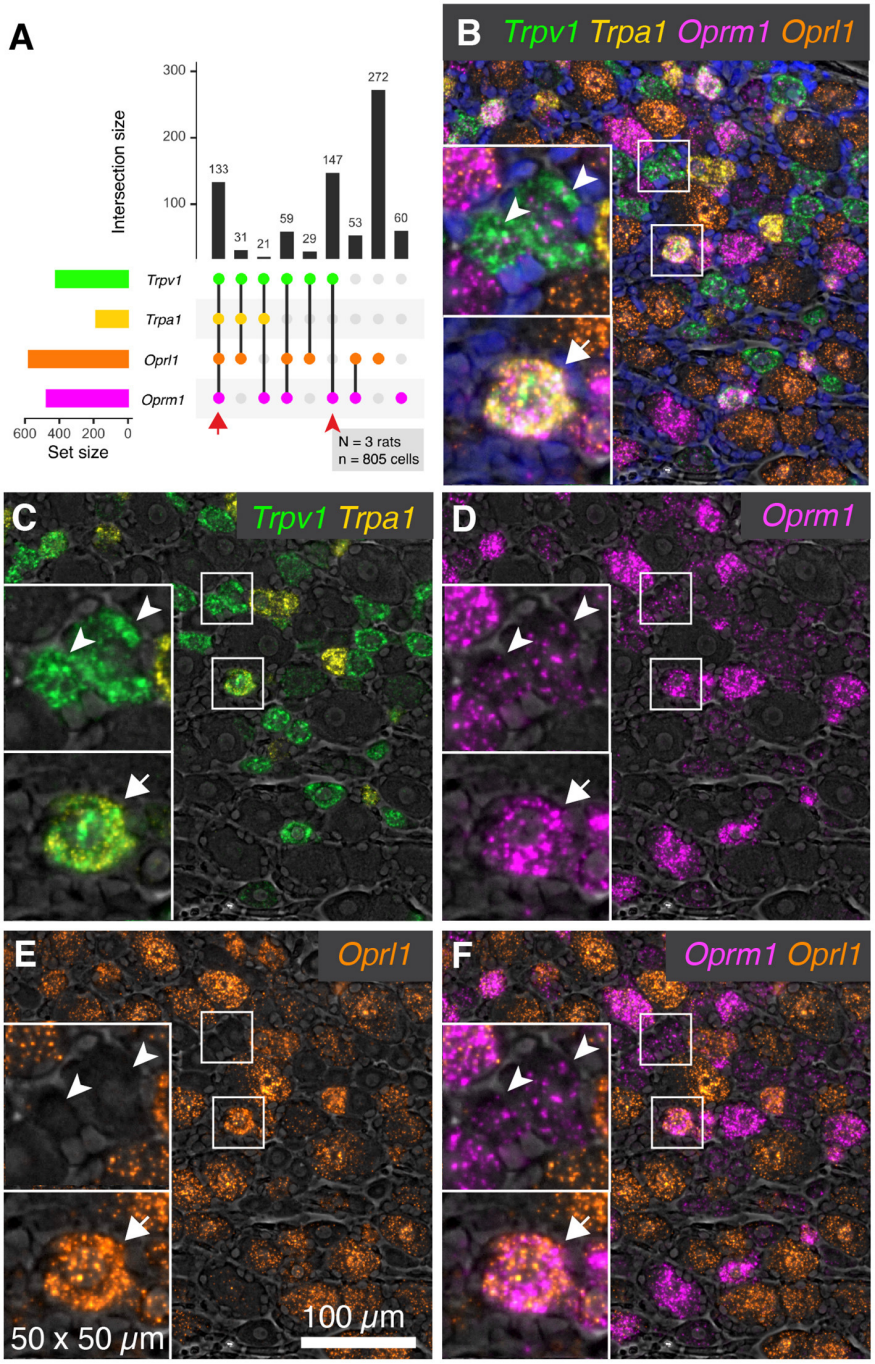
Co-expression patterns of nociceptive ion channels and opioid receptors. To assess the overlap between nociceptive transducing ion channels and opioid receptors, multiplex fluorescent hybridization was performed for *Trpv1, Trpa1, Oprm1* and *Oprl1*. This analysis was performed in *N* = 3 rats total, and 805 cells. **(A)** Counts were tabulated for co-expression of these four markers in each cell, and the intersections of these are plotted. The most prevalent count group was *Oprl1* alone, due to the near-ubiquitous nature of this marker (272 cells). Cells positive for all four labels (133 cells) were also prevalent, indicating overlap between nociceptive ion channels (*Trpv1* and *Trpa1*) and the mu-opioid receptor *Oprm1* as well as *Oprl1*. Populations containing *Trpv1* and *Oprm1* with or without *Oprl1* (59 cells with all three and 147 cells with *Trpv1*/*Oprm1* only) were also prevalent, reinforcing the overlap between these two markers. **(B)** A representative panel showing a diverse field of cells is shown, with quad-positive neurons highlighted (arrows). Additionally, *Trpv1*+/*Oprm1*+ cells are indicated (arrowheads). **(C–F)** To increase the visibility of the highly multiplexed image, individual channels and channel pairs are shown for the image in **(B)**, with the channel identity found in the bottom right of each panel. **(C)** A panel showing only *Trpv1* and *Trpa1* was selected to show the relationship between these two nociceptive ion channels. **(D)** A range of intensities was observed for *Oprm1*. Note the presence of less prominent puncta in *Trpv1*+/*Oprm1*+ cells (arrowheads). **(E)** *Oprl1* alone. **(F)** *Oprm1* and *Oprl1*. Scale bar applies to all images.

RNA-Seq of rat dorsal root ganglia showed modest expression of *Oprm1* and comparatively higher expression of the opioid-related nociceptin receptor 1 (*Oprl1*) gene (7.9 and 25.8 sFPKM respectively) (Sapio et al., 2016). This receptor binds the opioid-like heptadecapeptide nociceptin, derived from the pronociceptin precursor and has sequence similarity (Phe-Gly-Gly-Phe-Thr-Gly-Ala-Arg-Lys-Ser-Ala-Arg-Lys-Leu-Ala-Asn-Gln) to dynorphin A1-17 (Tyr-Gly-Gly-Phe-Leu-Arg-Arg-Ile-Arg-Pro-Lys-Leu-Lys-Trp-Asp-Asn-Gln). To assess the overlap between expression of *Oprm1* and *Oprl1* and the nociceptive transducing ion channel genes *Trpv1* and *Trpa1*, multiplex fluorescent *in situ* hybridization was performed for these four transcripts together. The plot in Figure 4A is derived from 805 cells, counted in 3 sections from 3 animals. Co-expression was assessed for each of the 4 genes. The most prevalent neuronal type identified by these four labels was *Oprl1* alone neurons, which accounted for 272 cells (33.8%). When coexpression was taken into account, *Oprl1* was observed to be expressed in a majority of DRG neurons (71.6%). Cells positive for all four labels (133 cells, 16.5%) were also among the most prevalent co-expression combinations, indicating a strong overlap between the nociceptive ion channels *Trpv1* and *Trpa1* with opioid receptor genes *Oprm1* and *Oprl1*. Populations containing the nociceptive marker gene *Trpv1* and *Oprm1* with or without *Oprl1* (59 cells with all three and 147 cells with *Trpv1*/*Oprm1* only) were also prevalent, reinforcing the overlap between this thermal nociceptive ion channel and the main opioid analgesia-producing receptor. A representative panel of neurons is shown in Figure 4B, with neurons positive for all 4 labels indicated with arrows, and *Trpv1*+*/Oprm1*+ neurons indicated with arrowheads. These arrows/arrowheads are maintained in all four channels to show individual signal. The overlap between the two nociceptive ion channel genes (*Trpv1* and *Trpa1*) is visualized in Figure 4C. Within these populations, a wide range of signal intensity was detected for *Oprm1* indicating variable expression of the μ-opioid receptor gene in the populations described with this 4-plex labeling scheme. In the visualization of *Oprl1* alone, note the broad labeling of many primary afferent neurons (Figure 4E). Analogous to the visualization in Figure 4C, the two opioid receptors (*Oprm1* and *Oprl1*) detected in this staining panel were visualized together in Figure 4F. Note the incomplete overlap between these two labels.

### Quantitative Co-expression Analysis for the Nociceptive Ion Channel *Trpv1* and *Trpa1* and Opioid Receptor *Oprm1* and *Oprl1* Genes

**Figure 5:**
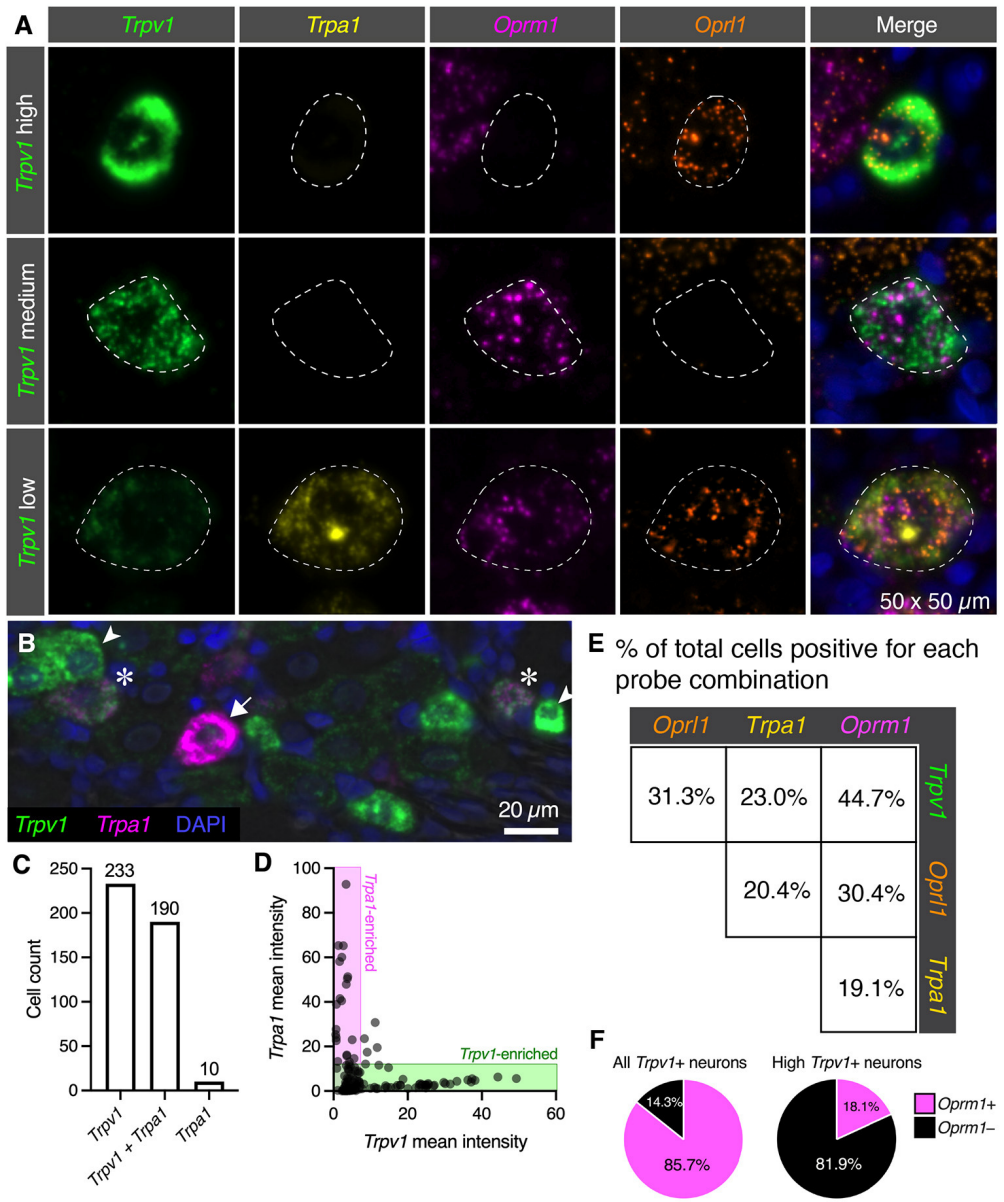
Quantification of nociceptive ion channel and opioid receptors. We further examined the subpopulations of DRG neurons shown in Figure 3. **(A)** Enlarged and enhanced fields were shown for each of three representative cell populations corresponding to high, medium and low *Trpv1* expression. Note that several populations of cells have medium *Trpv1* expression, but that one of the most common subtypes (*Trpv1* and *Oprm1* alone), was selected. Similarly, a quad-positive neuron was selected as representative of low *Trpv1* expression. **(B)** To examine the relationship between *Trpv1* and *Trpa1* in particular, expression levels in perikarya of cells expressing either marker were examined in further detail. In this representative field, neurons strongly enriched for *Trpv1* (arrowheads) or *Trpa1* (arrow) are indicated, as well as two neurons (asterisks) that co-express these two ion channel genes. **(C)** Using cell counting, we identified the coincidence of these two markers. More cells express *Trpv1* than *Trpa1* with the majority of *Trpa1*+ cells co-expressing *Trpv1*, and only 10 *Trpa1*+ cells expressing no detectable *Trpv1*. **(D)** However, cells expressing high levels of either *Trpv1* or *Trpa1* express very low levels of the other transcript. Therefore, the expression of these two markers while coincident, appears to be anticorrelated (note that points fall on the axes rather than in the middle of the plot. **(E)** Pairwise coincidence is shown for each of the four labels in this experiment. All of these combinations are common (with the rarest being approximately 19.1% of all cells). High *Trpv1* neurons, in particular, seemed to differ from other *Trpv1* neurons in terms of *Oprm1* expression. **(F)** We addressed this by counting high *Trpv1* neurons separately from other *Trpv1* neurons (shown in pie charts). Whereas average *Trpv1* neurons expressed *Oprm1* the majority of the time (85.7%), the high Trpv1 neurons expressed *Oprm1* only 18.1% of the time. On average, the expression in these cells was also lower (Supplementary Figure 3). Outlines (dotted lines) of neuronal perikarya are shown to enhance visbility.

Individual neuronal perikarya of three distinct populations were examined by multiplex fluorescent *in situ* hybridization (Figure 5A). The high *Trpv1* expressing neurons were selected out for separate analysis based on their unique signature of *Trpv1* expression (see Figure 2). The major populations colocalizing *Trpv1*+/*Oprm1*+ were low and medium expressing *Trpv1* neurons (middle row, Figure 5A *Trpv1* medium). Similarly, while not all low *Trpv1* expressors are positive for all four labels, we focused on such quad+ neurons for visualization (bottom row, Figure 5A *Trpv1 low*). This is the population that coexpressed mRNA encoding the chemo-nociceptive ion channel *Trpa1*. As we observed that the cells labeled with all four markers were generally low for *Trpv1*, we examined the relationship between *Trpv1* and *Trpa1* in greater detail. In the representative field (Figure 5B), we observed that *Trpv1* and *Trpa1* were generally not strongly expressed in the same cells, although some co-positive cells were observed with low levels of both markers. Note the cells strongly expressing *Trpv1* (arrowheads) or *Trpa1* (arrows), as well as cells co-expressing both markers (asterisks). Cell counts by marker positivity were performed to evaluate the findings of our peripheral ganglionic comparison study between the nodose, DRG and superior cervical ganglia (Sapio et al., 2020b) (Figure 5C). We found that the most prominent populations of cells were *Trpv1*+/*Trpa1-* (*Trpa1*-; 233 cells, 53.8%) followed closely by the faintly labeled *Trpv1*+/*Trpa1*+ (190 cells, 43.9%) population. The *Trpv1*-/*Trpa1*+ cells (10 cells, 2.3%) comprised the rarest subgroup. In a quantitative analysis of expression levels, *Trpv1* and *Trpa1* intensity measurements tended toward the axes (Figure 5D, shaded areas), indicating an inverse correlation with expression levels. Note that no cells are identified in the center of the graph, which would indicate bright co-expression of these two markers. Pairwise coincidence is shown for each of the four labels in this experiment (Figure 5E). All of these combinations are common (with the rarest being approximately 19.1% of all cells) for *Trpa1* and *Oprm1*. In these analyses, we tried to identify differences among the high *Trpv1*+ population, and noticed that they seemed less likely to express high levels of *Oprm1*. This was formally quantified, and is displayed in pie charts (Figure 5F) showing that the subgroup of *Trpv1*+ high-expressing neurons (*N* = 33) expressed *Oprm1* only 18.1% of the time, whereas *Trpv1*+ neurons overall expressed *Oprm1* 85.7% of the time. We also noted that these cells expressed a low level of *Oprm1* on average (Supplementary Figure 3).

### Heterogeneous Levels of *Trpm8* mRNA in Rat DRG and Co-expression Analysis With *Trpv1*

**Figure 6:**
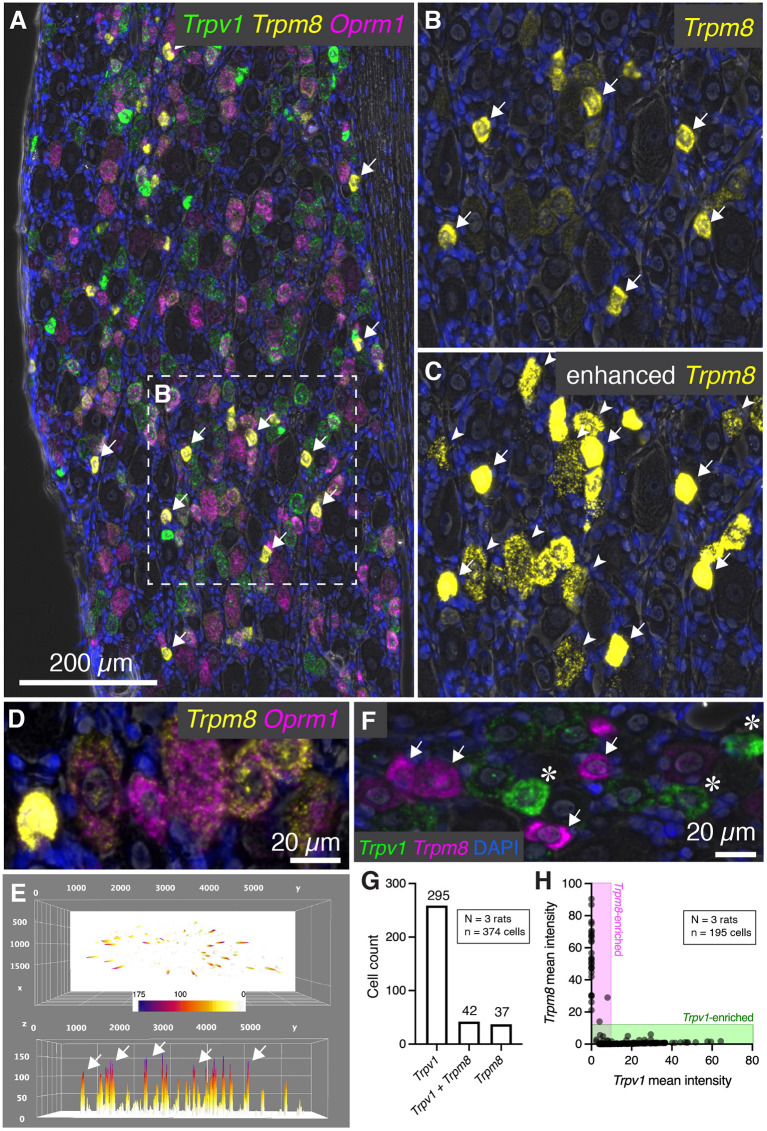
Characterization of *Trpm8* expression levels in rat DRG. To assess the overlap between hot and cold thermal nociceptive transducing ion channels, multiplex fluorescent hybridization was performed for *Trpv1*, and *Trpm8* alongside *Oprm1*. **(A)** Panoramic view of triple-labeled rat DRG. **(B)** Cells positive for *Trpm8* were brightly labeled small diameter neurons (white arrows). However, a number of other neurons were also visible with lower levels of *Trpm8*. **(C)** Similar to what was performed for *Trpv1*, enhancement of the image saturates the high-expressing *Trpm8* neurons, but allows for visualization of the much lower intensity staining observed in other neurons. Note that these neurons still have clear punctate staining pattern and that this staining pattern was reproduced with a different probe for confirmation (Supplementary Figure 4). **(D)** Another characteristic of *Trpm8* neurons is that 100% of them co-express *Oprm1* to some extent. This relationship is visualized in the representative field. **(E)** A surface plot of *Trpm8* expression levels is shown with arrows pointing to the highest expressing *Trpm8*+ neurons. Note the lower peaks span a range of expression levels. **(F)** The relationship between *Trpv1* and *Trpm8* was further explored by quantifying levels of expression of these two thermal transducing ion channels shown in the representative field. **(G)** The *Trpv1*+ population was much larger than the *Trpm8*+ population, and approximately half of *Trpm8*+ neurons co-expressed *Trpv1*. **(H)** However, with quantification we saw that no cell co-expressed these markers strongly, and that their expression was anticorrelated (note that the points fall on the axes rather than in the center of the plot).

In addition to *Trpv1*, several other temperature sensitive ion channels are implicated in noxious stimuli transduction and nociceptive signaling in baseline and disease states (reviewed in Koivisto et al., 2022). These ion channel genes are useful markers of nociceptive cell populations and have been targeted for therapeutic interventions. While Trpv1 responds to temperatures in the warm to hot temperature range, another ion channel, Trpm8 is activated by cold temperatures and participates in cold allodynia (Bautista et al., 2007; Knowlton et al., 2010). Multiplex fluorescent *in situ* hybridization revealed heterogeneous expression of the *Trpm8* mRNA, with most of the signal concentrated within sparsely distributed, small diameter neurons (Figure 6A, arrows). The results for *Trpm8* expression were similar to what was observed for *Trpv1* in Figure 2, showing a range of expression consisting of a small subpopulation of brightly labeled neurons and a second set of neurons with weaker signal. An enlarged visualization of *Trpm8* alone (Figures 6B,C) showed the densely labeled small to medium sized neurons (arrows), as well as additional neurons showing less intense labeling (arrowheads). The convergence of transcripts was examined using multiplex fluorescent *in situ* hybridization with four labels (*Trpv1, Trpm8*, and *Oprm1*). This staining combination revealed that virtually all *Trpm8*+ neurons co-expressed *Oprm1*, albeit at a range of expression values as seen in the representative field (Figure 6D). A surface plot was generated to visualize the range of expression profiles in the DRG neurons. Note that the high expressors appear as isolated peaks in this view, indicating that they are quantitatively separable from other *Trpm8*+ neurons (Figure 6E). Also note that the lower expressing *Trpm8*+ neurons required enhancement for visualization, indicating they express *Trpm8* at a much lower level than the high-expressing neurons. This lower expression was validated by using a second *Trpm8* probe to rule out origination from nonspecific signal (Supplementary Figure 3).

We further examined the co-expression pattern of the hot and cold thermosensory ion channel transcripts *Trpv1* and *Trpm8*, respectively. In the representative image (Figure 6F), densely labeled *Trpm8*+ (arrows) and *Trpv1*+ neurons (asterisks) are indicated. Co-expression analysis showed that the most prominent population was the *Trpv1*+/*Trpm8*- population (295 cells, 76.0%), as *Trpv1* is more broadly expressed than *Trpm8* (Figure 5G). The *Trpv1*+/*Trpm8*+ co-positive cells (42 cells, 10.8%) and *Trpm8*+ populations (37 cells, 9.5%) were less abundant, with approximately half of *Trpm8*+ cells coexpressing *Trpv1*. Similar to what was shown for *Trpv1* and *Trpa1*, fluorescence intensities of *Trpm8* vs. *Trpv1* signals tended to plot along the axes (shaded areas, Figure 6H) indicating that strong expression of either marker gene was observed mainly in separate sensory neurons (cell quantification from N=3 rats). This indicates that strongly *Trpv1*+ neurons never strongly expressed *Trpm8*, and strongly *Trpm8*+ neurons never strongly express *Trpv1*.

### Co-expression Analysis of the Thermal Nociceptive Ion Channels *Trpv1* and *Trpm8* With the μ-Opioid Receptor (*Oprm1*) and the Voltage-Gated Ion Channel Subunit *Scn11a*

**Figure 7:**
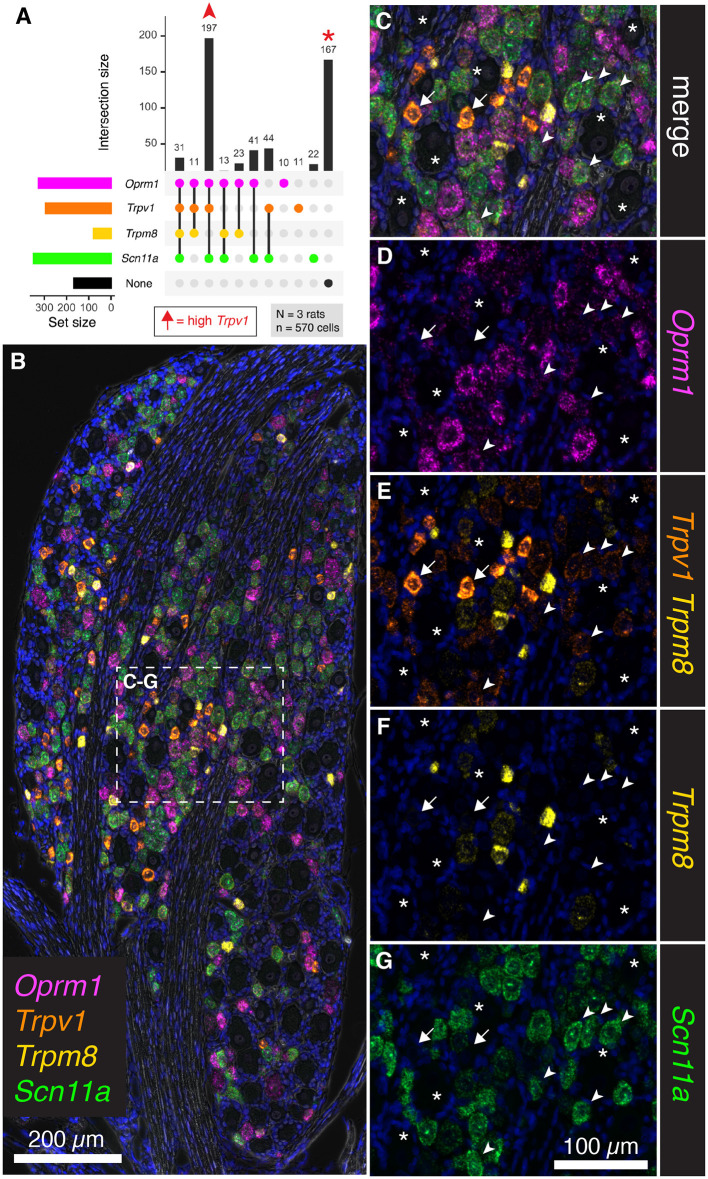
Co-expression analysis for *Trpv1, Trpm8, Oprm1*, and *Scn11a*. To further assess the overlap between nociceptive transducing ion channels and opioid receptors, multiplex fluorescent hybridization was performed for *Trpv1, Trpm8, Oprm1* and *Scn11a* in *N* = 3 rats (*n* = 570 cells). In this experiment, we investigated the mRNA coding for the cold-pain transducing nociceptive ion channel Trpm8 as well as the mRNA encoding the voltage-gated ion channel subunit Scn11a. **(A)** Counts were tabulated for co-expression of these four markers as before. The most prevalent group were neurons co-positive for *Trpv1, Oprm1* and *Scn11a* (197 cells). Notably, a large number of neurons was also unlabeled (167 cells). Arrows in the plot in **(A)** are used to identify cells in the representative fields in subsequent panels. High *Trpv1* neurons (arrows) are not in the plot in **(A)**, but were highlighted in the representative fields. **(B)** A panoramic stitched image is shown to demonstrate the anatomical location of these markers within the greater structure. In the center of the field, a representative area was enlarged for subsequent panels. **(C)** Overlay of the 4-plex label with DAPI and brightfield is shown. Note that examples of the most prevalent cell type, *Trpv1*+/*Oprm1*+/*Scn11a*+ neurons are indicated (arrowheads). Larger diameter cells with no reactivity for these markers are indicated (asterisks). A singular putative thermosensing c-fiber is indicated with very high levels of *Trpv1* (arrow). **(D)**. Note the varying levels of *Oprm1* in *Trpv1*+/*Oprm1*+/*Scn11a*+ neurons (arrowheads), and absence of Oprm1 in the high-expressing Trpv1+ neuron (arrow). **(E)** In an overlay of *Trpv1* and *Trpm8*, note that cells were generally high in one or the other but not both markers. **(F)** Also note the sparse labeling of *Trpm8*, indicative of its specialized function. **(G)** The sodium channel subunit Scn11a was prevalent in *Trpv1*+/*Oprm1*+/*Scn11a*+ neurons (arrowheads), as well as strongly expressed in many cells throughout the ganglion. Scale bar applies to all images. Scale bar applies to **(C–G)**.

To further assess the overlap between nociceptive transducing ion channels and opioid receptors, multiplex fluorescent *in situ* hybridization was performed for the thermally responsive ion channels *Trpv1* and *Trpm8*, in conjunction with the μ-opioid receptor gene *Oprm1*. An additional marker of nociceptive neurons, the voltage-gated sodium channel subunit (Na_V_1.9) gene, *Scn11a*, was also included. Human mutations in *SCN11A* cause pain insensitivity or episodic pain syndromes depending on the nature of the mutation (Leipold et al., 2013; Zhang et al., 2013). The total number of cells counted was 570 from *N* = 3 rats. Upon quantification of co-expression of these four markers, the most prevalent subgroup was composed of neurons co-positive for *Trpv1, Oprm1* and *Scn11a* (197 cells 34.6%, Figure 7A). Notably, many neurons were also unlabeled (167 cells 29.2%). In a panoramic visualization (Figure 7B) of these cell populations, a representative field was chosen to highlight the staining patterns. Examples of the most prevalent cell type (*Trpv1*+/*Oprm1*+/*Scn11a*+) are indicated with arrowheads (Figures 7C–G). Large diameter cells with no reactivity for these markers are also indicated (asterisks). A putative thermosensory C-fiber with very high levels of *Trpv1* is indicated (arrow). This type of cell does not show reactivity for the other three markers, indicating minimum co-localization of either nociceptive or analgesic markers in the highest expressing *Trpv1*+ neurons. Note the varying levels of *Oprm1* in *Trpv1*+/*Oprm1*+/*Scn11a*+ neurons (arrowheads), which could indicate heterogeneity in this labeled class. In an overlay of *Trpv1* and *Trpm8* (Figure 7E), note that cells were generally high in one *or* the other but not both markers indicating the specialized role of *Trpm8* in cold thermonociception and cold pain, and its localization within a small, discrete subclass of sensory afferents in the rat. Consistent with its role in nociception, the sodium channel subunit *Scn11a* was prevalent in *Trpv1*+/*Oprm1*+/*Scn11a*+ neurons (arrowheads), as well as strongly expressed in many cells throughout the ganglion. We also considered the *Trpm8*+ cell populations quantitatively (Supplementary Figure 4), showing that high/medium *Trm8*+ cells were negative for *Trpv1* and *Scn11a*, whereas low expressing *Trpm8*+ cells have low levels of these markers.

### Evaluation of *Trpv1, Oprm1, Oprl1* and the Proprioceptive Neuron Marker Secreted Phosphoprotein 1, Osteopontin (Spp1)

**Figure 8:**
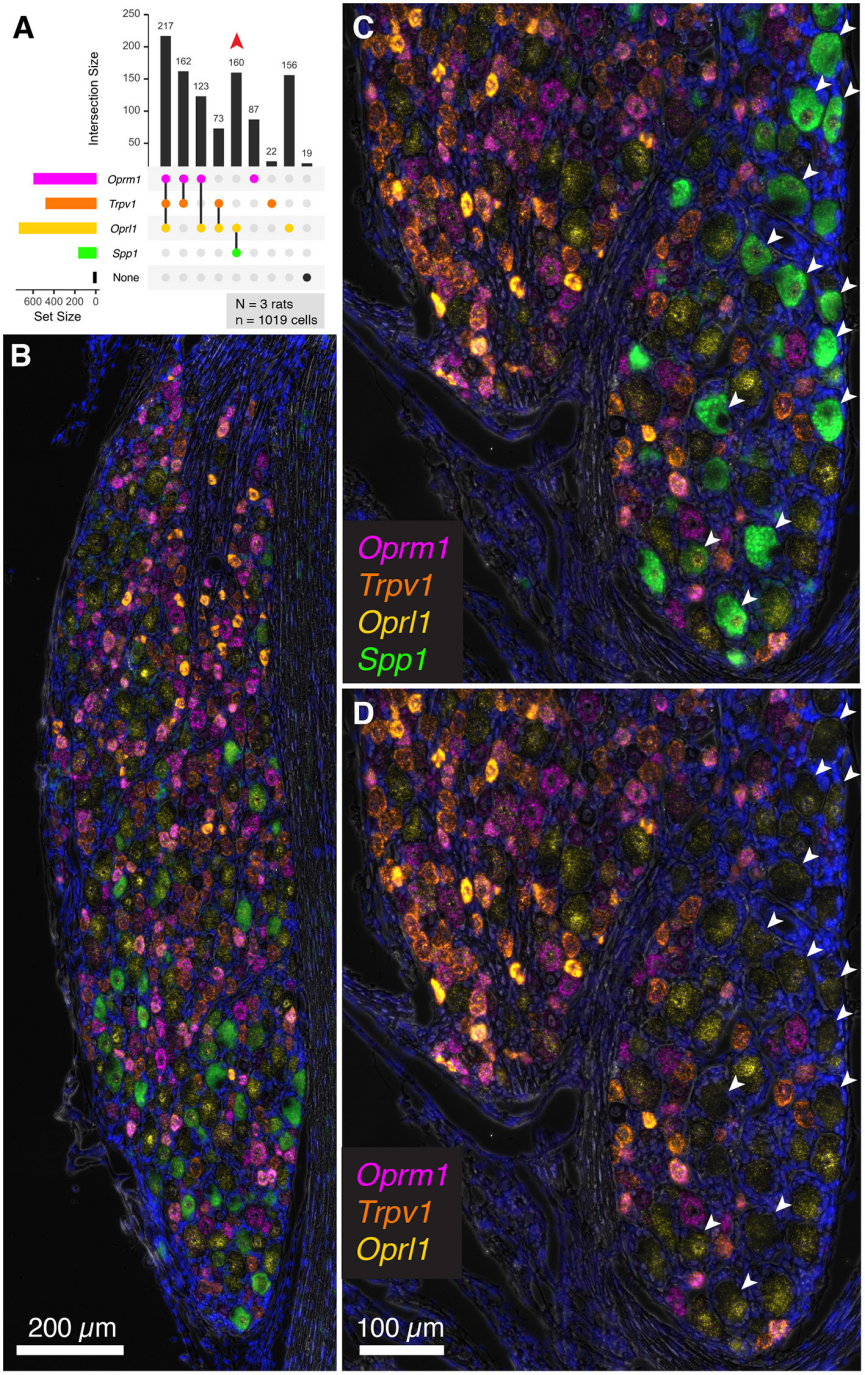
Co-expression of the nociceptive and analgesic markers *Trpv1, Oprm1*, and *Oprl1* with Secreted Phosphoprotein 1/Osteopontin (*Spp1*) transcript. Co-expression of *Trpv1, Oprm1*, and *Oprl1* with *Spp1*, a marker of DRG neurons thought to be chiefly implicated in proprioception. **(A)** In a co-expression plot, all of the *Spp1*+ neurons co-expressed *Oprl1*, while none showed expression of *Trpv1* or *Oprm1* (83 *Spp1*+/*Oprl1*+ neurons, red arrow). **(B)** A panoramic view of a whole rat DRG stained for this combination of markers. Note the distinct signal for *Spp1*. **(C)** An enlargement of a different DRG neuron shows spatially distinct areas with and without *Spp1* neurons. **(D)** Note that *Spp1*+ neurons (arrows) do not contain any *Trpv1* or *Oprm1* signal.

To extend the multiplex fluorescent *in situ* hybridization staining experiments to include a non-nociceptive marker, we examined co-expression of algesic and analgesic markers with osteopontin (*Spp1*), which labels large diameter neurons (with an average diameter of approximately 38 μm). These neurons are thought to be chiefly implicated in proprioception (Ichikawa et al., 2000; Usoskin et al., 2015; Saito-Diaz et al., 2021). In the co-expression plot in Figure 8A, all of the *Spp1*+ neurons co-expressed the nociceptin receptor *Oprl1*, while none showed expression of *Trpv1* or *Oprm1* (160 *Spp1*+/*Oprl1*+ neurons, red arrow). In a panoramic view of whole rat DRG, *Spp1*+ neurons constitute a distinct population (Figure 8B). Note also that these neurons tended to be spatially arranged in a particular area of the DRG section (Figures 8C,D).

### Summary Figure of Identified Neurons and Their Properties

**Figure 9:**
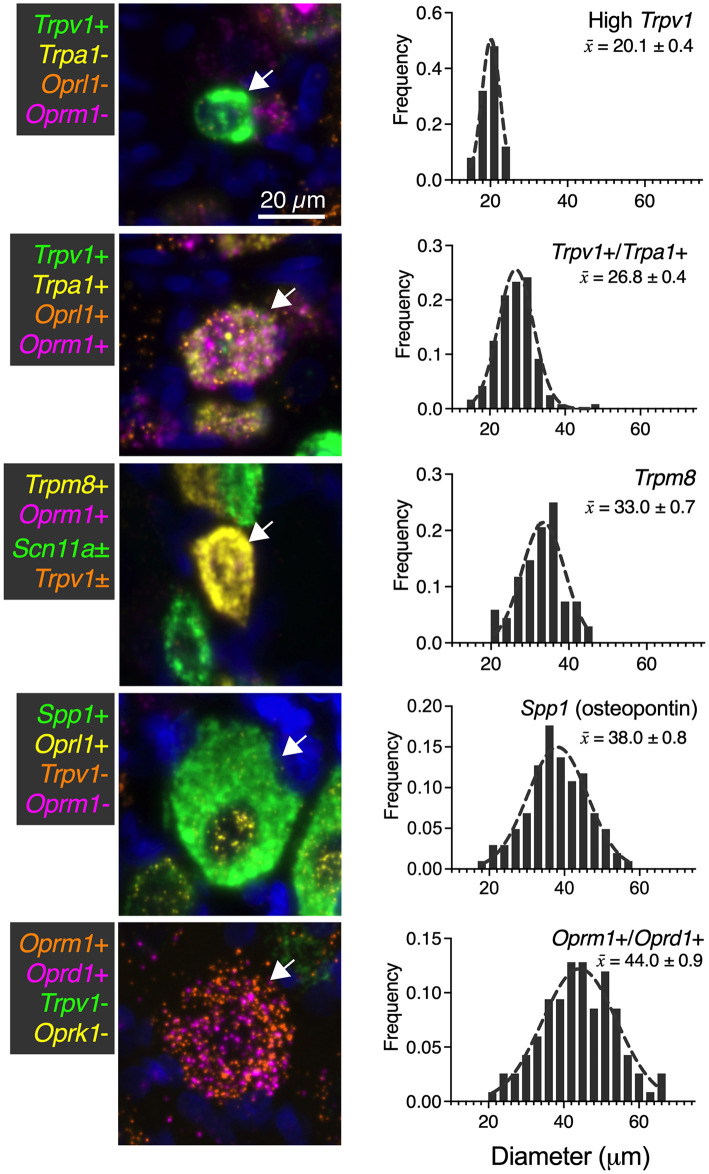
Summary of identified cell types with size analysis. For each of the major identified cell types in the present study, a representative image (66 μm square) is shown. Additionally, histogram information of the cell size for each population is identified in ascending order of mean size ± SEM. High *Trpv1* neurons were the smallest cells (20.1 μm ± 0.4). *Oprm1*+/*Oprd1*+ neurons were the largest cells identified, with an average size of 44.0 μm ± 0.9. Scale bar applies to all images. These analyses were performed with at least *N* = 3 rats.

While numerous categories of co-expression are documented, five readily distinguishable neuronal groups are shown in Figure 9 with frequency distributions of their cell diameters. The high *Trpv1*-expressing neurons were the smallest in size with a 20 μm mean diameter (*n* = 25 cells). The *Trpv1*/*Trpa1* cell population (*n* = 120 cells; often “quad positive,” as in Figure 4; ~26.8 μm average diameter) contains two algesic-sensing ion channels and the μ-opioid receptor that can confer analgesia. This analysis was performed using the double label as a selection criteria, but note that a subpopulation of these cells (such as that in the representative image) has additional labels. The diameter of the high expressing *Trpm8* cells was on average 33.0 μm (*n* = 68 cells), indicating a larger diameter than high *Trpv1* neurons (21 μm) or *Trpv1*/*Trpa1* cells (26 μm). The largest neuron identified was the co-positive for the μ-δ opioid receptor (*Oprm1*+/*Ord1*+) neurons (44 μm average diameter). These were even larger than the osteopontin (*Spp1*) expressing proprioceptive neurons (38 μm average diameter; *n* = 102 cells). In mouse, the osteopontin immunoreactive neurons give rise to the spiral endings innervating muscle spindles (Ichikawa et al., 2000; Chiu et al., 2014).

We identified a significant difference in diameter between each of the groups (*p* < 0.05). Significance testing was performed between the five groups (all comparisons) using a Kruskal-Wallis test with Dunn's correction for multiple comparisons (Prism 9, GraphPad). These findings were calculated from the same sections as other *N* = 3 analyses in other figures, although sampling was expanded (*n* = 117 cells in Figure 9) for this analysis due to the rarity of the *Oprm1*+/*Oprd1*+ population.

## Discussion

This study examined rat primary afferent neurons using multiplex fluorescent mRNA *in situ* hybridization to understand nociceptive and analgesia mechanisms in the peripheral nervous system. We consider identifying critical neuronal populations essential knowledge for developing potential peripherally acting non-opioid analgesics that do not exhibit central nervous system side effects. The investigation focused on determining co-expression patterns of nociceptive-, and analgesia-transducing molecular markers in DRG neurons. We hypothesized the conjuncture of these two types of molecules in one or more neuronal subpopulations would provide a colocalization matrix that could be used to generate a molecularly informed approach to peripheral analgesia and analgesic drug development.

The populations of nociceptive *Trpv1*+ neurons were heterogeneous, expressing varying levels of *Trpv1* and the μ-opioid receptor *Oprm1*. Co-expression analysis indicated differing degrees of μ-opioid expression among the two populations of *Trpv1*+ neurons that we hypothesize subserve thermosensory and tissue-injury nociception. We identified a population of very high expressors of *Trpv1* that are absent of *Oprm1*. In contrast, most low and medium expressors of *Trpv1* do express *Oprm1*, and are the evident candidates for the neuronal population inhibited by μ-opioid agonist analgesics, especially when given intrathecally or epidurally, administration paradigms where the injectate bathes the central projection of dorsal root ganglion axons entering the spinal cord. The co-expression of *Trpv1* and *Oprm1* receptors within the same cell types provides both cellular and molecular level explanations for the efficacy of opioid agonists. That is, opioid drugs act by inhibiting the very cells responsible for transmitting certain types of nociceptive stimuli from the periphery to the central nervous system. Available evidence suggests that opioid analgesia occurs, in part, through presynaptic inhibition of transmitter release from *Trpv1*+ DRG afferent terminals in spinal cord and potentially spinal cord μ-expressing neurons (Kondo et al., 2005; Chen and Pan, 2006; Xanthos and Sandkühler, 2014; Che et al., 2021). Co-expression of *Trpv1* was never observed with *Spp1*, a marker for large diameter proprioceptive neurons, a finding that validates that nociception and proprioception are mediated by distinct neuronal populations. Additionally, the lower expressors of *Trpv1* co-expressed other markers implicated in pathological pain states. For example, Trpm8, a nociceptive ion channel implicated in cold thermosensation (Bautista et al., 2007) and cold allodynia (Xing et al., 2007) was identified in some lower-expressing *Trpv1* sensory neurons. Additionally, the ion channel NaV 1.9 (encoded by *SCN11A*), whose gain-of-function mutation leads to familial episodic pain (Leipold et al., 2013), was also identified in the low to medium-expressing *Trpv1* population. In contrast, the small diameter high *Trpv1* expressing neurons were negative for all of these markers, most likely indicating an exclusive role as a dedicated labeled line for thermonociception and/or non-noxious thermosensation (Kobayashi et al., 2005; Craig, 2018). Coexpression analysis is also a critical step for gaining insight into the algesic and analgesic receptors on DRG neurons, and could be important for understanding indirect interactions between painful and analgesic target receptors. In particular, there are established interactions between pain transducing ion channels and opioid receptors (Chew et al., 2019).

The μ-opioid receptor is one of the most clinically important targets for analgesia, with the majority of highly impactful orally and intravenously available analgesic agents acting through this receptor. While there are three canonical opioid receptors (μ, δ, and κ), and a fourth opioid-like paralog (*Oprl1*), only μ-receptor agonists are used clinically at this time, although extensive medicinal chemical and preclinical development on the other receptors has occurred or is in progress (Calo et al., 2000; Floyd et al., 2009; Bardoni et al., 2014; Viscusi et al., 2021). Additionally, some controversy exists over detection of these receptors, as they are frequently expressed to a low degree and difficult to quantify accurately (Imlach and Christie, 2014; Iadarola et al., 2015; Sapio et al., 2016). In this regard, the present study fills a critical knowledge gap by delineating the co-expression matrix of these receptors in detail. We characterize the expression pattern of all four receptors, and, in particular, the canonical three together. One point of contention is the pharmacological relevance of opioid receptor heterodimers. In order for the heterodimer to form (Jordan and Devi, 1999; Erbs et al., 2015; Gaborit and Massotte, 2021), both receptors would, at a minimum, have to be expressed in the same cell. Our data confirm that co-expression of the μ-, δ- and κ-opioid receptors does indeed occur in some combinations, albeit in a very small population of neurons. This heterogeneity is confirmed by recording and staining from rat DRG neurons, although some differences in proportions of cells between the present study and the *in vitro* recording investigation are seen and are likely due to methodological differences (Rau et al., 2005). Interestingly, some large cells were observed with high concentrations of μ- and δ-opioid receptors (Figure 1E), consistent with putative mechanosensory nociceptors, based on genetic anatomical tracing studies done in the mouse (Bardoni et al., 2014). These anatomical findings showing colocalization of *Oprm1* and *Oprd1* may be related to the observed analgesic synergy between μ- and δ-opioid agonists in the periphery (Bruce et al., 2019). Another study using genetic labeling in mice showed that the κ-opioid receptor was frequently expressed in myelinated lanceolate or circumferential nerve endings around hair follicles, and demonstrated that κ-opioid receptor function at these afferents modulated pain and itch (Snyder et al., 2018). In our rat study the majority, although not all κ-opioid receptor expressing neurons, also expressed the μ-opioid receptor indicating that this pair of receptors likely functions in the same cell to modulate some modalities of nociception.

Another opioid-like receptor examined in this study is the nociceptin/orphanin FQ receptor (NOP receptor, encoded by *Oprl1*). This is the least studied of the four opioid-like receptors, and although it has been proposed to have multiple avenues toward clinical potential (Lambert, 2008), none have been realized to date. One finding that is particularly relevant to pain and analgesia, is the observation that intrathecal administration of nociceptin, the endogenous agonist at the NOP receptor was analgesic, and this analgesia was not naltrexone reversible, suggesting NOP-dependent analgesic actions are distinct from analgesia mediated at other opioid receptors (Ko et al., 2006). In our staining, we identified signal for *Oprl1* expression in almost all DRG neurons, which does not exclude its utility as an antinociceptive agent, but suggests very broad function(s), and may result in off target side effects on non-nociceptive fibers. Notably, broad action, or expression of an analgesic target outside of the nociceptive neurons is not necessarily indicative of reduced analgesic potential, but the ubiquity of *Oprl1* signal does contradict the supposition that this receptor is involved in endogenous analgesia in a specific fashion.

One of the key nociceptive markers examined in the present study is Trpv1. This receptor is the endogenous receptor for capsaicin, the pungent ingredient in hot peppers and resiniferatoxin, a new analgesic currently undergoing clinical trials (NCT03542838, NCT02522611, NCT00804154, NCT04885972, and others) (Caterina et al., 1997). It has long been appreciated that *Trpv1* is expressed in several subclasses of neurons, and more recently these populations have been described to a greater extent in several species (Goswami et al., 2014; Isensee et al., 2014; Usoskin et al., 2015; Sapio et al., 2018; Tavares-Ferreira et al., 2022). The overall pattern of expression of *Trpv1* is similar to previous studies, although it appears that the RNAScope procedure, in particular, labels more neurons than most antibody-based approaches (Mitchell et al., 2010, 2014; Goswami et al., 2014; Poulson et al., 2020; Sapio et al., 2020b; Shiers et al., 2020; Hall et al., 2022; Tavares-Ferreira et al., 2022). It is also notable that the percentage of Trpv1+ neurons as assessed by a technique as sensitive as RNAScope is not necessarily identical to the number of capsaicin-responsive neurons. For example, the percentage of neurons killed by capsaicin incubation in culture is estimated to be approximately 37% in one study (Wood et al., 1988), which is also similar to the number of cultured mouse lumbar DRG neurons responding to 300 nM capsaicin (39%) (Teichert Russell et al., 2012). Therefore, it appears that at a certain level of expression is required to produce robust enough calcium influx to compromise cellular integrity (Karai et al., 2004b). That is, approximately half of the neurons detected as *Trpv1*+ in our study (presumably the most strongly labeled neurons) are likely to be capsaicin responsive.

Generally, it has been observed that there is a small number of densely immunoreactive small diameter Trpv1+ neurons, as well as a larger number of larger diameter and less densely stained neurons (Tominaga et al., 1998; Cavanaugh et al., 2011b; Goswami et al., 2014). Presumably, this encompasses at least two functionally defined TRPV1+ subclasses: the Trpv1+ C-fibers and A-δ fibers (Mitchell et al., 2010, 2014). The molecular identity of the TRPV1+ A-δ fibers is still being determined (Raithel et al., 2018), in part due to the small percentage of A-δ neurons in DRG (Lawson et al., 2019). Our study suggests that the small diameter high expressing *Trpv1*+ fibers in the rat contain a narrower repertoire of noci-responsive TRP channels, potentially implicating a more exclusive thermal nociceptive role which, at least in the rat, does not appear to be sensitive to opioid-induced analgesia. Differential thermal activation of A-δ and C-fibers suggest that, based on behavioral withdrawal, the C-fiber population is the one that is less sensitive to μ-opioid receptor agonists (Mitchell et al., 2014) and the A-δ neurons are a subpopulation of the medium to low TRPV1 expressing neurons. To some extent, a similar phenomenon has been examined in humans in a study using high dose transdermal capsaicin patch. In this study, sensitivity to noxious (55°C) thermal stimulation was confined to the capsaicin-treated region, while intensity ratings for less noxious (44°C) or non-noxious (38°C) thermal stimuli were *reduced* distal to the patch-treated skin (Van Neerven and Mouraux, 2020). This is potentially consistent with the idea that higher expressing *Trpv1*+ fibers are more capsaicin sensitive and primarily involved in thermonociceptive sensation, while the more broadly, polymodal nociceptive fibers may have less *Trpv1* but co-express multiple noci-responsive channels. However, notably, this type of experiment may also be influenced by other variables such as receptive field size and central modulatory effects. Further studies will be needed in humans to tease out the function of these Trpv1+ populations.

Another TRP channel *Trpa1* was examined in regard to both *Trpv1* and analgesic-related opioid receptors. Trpa1 is the receptor for chemical nociception and is responsible for transducing stimuli such as allyl isothiocyanate, the active pungent ingredient in foods such as wasabi (Jordt et al., 2004; Mcnamara et al., 2007). In our previous study using transcriptomics and multiplex fluorescent *in situ* hybridization, we examined the expression pattern of *Trpv1* and *Trpa1* in DRG and nodose ganglion sensory afferents (Sapio et al., 2020b). In that study, we found that *Trpv1* and *Trpa1* were more highly co-expressed in nodose, whereas in the DRG, neurons that were high in either *Trpv1* or *Trpa1* tended to be low for the other, suggesting a separate, although not exclusive set of sensory pathways for thermal and chemical nociception in the peripheral nervous system. The present study confirms this result (Figure 6). The largely differential expression is also consistent with previous studies showing partial depletion of Trpa1 with Trpv1 agonists (Pecze et al., 2009) or coexpression of these receptors. However, *TRPA1* is apparently expressed in low expressing *TRPV1* population, which could explain why these neurons are resistant to TRPV1 agonist actions and could also explain the failure to deplete *TRPA1*-expressing neurons with TRPV1 agonists in some experiments (Isensee et al., 2014; Sapio et al., 2018, 2020b). The functional implication of co-expression of these receptors is being investigated. For example, heteromers have been reported between these two ion channels with different pharmacologic and biophysical properties and have been proposed to be involved in nociceptive sensitization (Fischer et al., 2014; Patil et al., 2020). Cells with both *Trpv1* and *Trpa1* also tended to have moderate to high amounts of *Oprm1* expression (see Figure 3, Quad+ neurons), consistent with their proposed dual nociceptive and analgesia-conferring properties (Akopian, 2011).

Trpm8 is the primary receptor for cold and cold pain (Bautista et al., 2007) and serves as a marker for cold-sensing sensory afferents (Renthal, 2020). We describe populations of high and low *Trpm8* expression, where presumably the high *Trpm8* expressing cells are those that have primarily been described in previous studies as cold thermosensory afferents (Le Pichon and Chesler, 2014; Jankowski et al., 2017), and the lower levels of *Trpm8* in other cells are currently of unknown significance. However, these could be polymodal neurons, or cells that become recruited at colder temperatures and/or during cold allodynia. Notably, while *Trpm8*+ afferents have been shown to be involved in responding to environmental cooling, a small population of *Trpm*8+ fibers has been reported to respond to noxious mechanical stimulation (Jankowski et al., 2017), and it has been suggested that in injury states a subpopulation of *Trpm8*+ fibers may become responsive to additional noxious modalities. Additionally, the processes underlying thermal encoding in the DRG can be complex, with populations of cells responding in a graded fashion to various temperatures (Wang et al., 2018). Furthermore, electrical recordings have shown that cooling-responsive cells comprise only about 2.3% of all DRG neurons (Lawson et al., 2019), whereas the total number of *Trpm8*+ cells we quantified was much larger than this estimate. While examining the full range of thermal-encoding cells is beyond the scope of this study, the coincidence of expression of *Trpv1* and *Trpm8*, which encode two major encoding ion channels of temperature, may be useful information for future investigations. This is also an indication that we are examining multiple classes of DRG neurons among the *Trpm8*+ population. This is one area where additional electrical characterization of these subtypes would be informative, as this can elucidate functional response characteristics to further segregate the DRG afferents (Petruska et al., 2000; Rau et al., 2005; Lawson et al., 2019). This is also interesting given that *Trpm8* expression level may be related to tuning thermal responsiveness, which is notable given the finding that various channels and/or channel combinations may be involved in thermal coding based on mouse studies (Paricio-Montesinos et al., 2020).

The overlap between *Trpm8* and *Oprm1* expression in the rat makes sense given reduction in clinical responses to cold sensation upon morphine (a mu-opioid receptor agonist) administration (Cleeland et al., 1996). This is also corroborated by the finding that morphine alleviated cold allodynia in rat models of chronic pain (Erichsen et al., 2005). Both cases support the idea that *Trpm8*+ cells are inhibited by μ-opioid receptor agonists. The *Trpm8*+ neurons also appear to be largely *Trpv1*-, indicating discrete types of peripheral neurons for the detection of hot and cold. This lack of overlap between *Trpv1* and *Trpm8*, is consistent with previous evidence demonstrating that *Trpm8*+ neurons are (in general) a small-diameter, relatively rare *Trpv1*- population of sensory afferents (Dhaka et al., 2008; Pecze et al., 2009; Le Pichon and Chesler, 2014; Jankowski et al., 2017). Although we did detect *Trpm8*+/*Trpv1*+ neurons, the expression was largely anticorrelated (Figure 6H) indicating that these two receptors are never strongly co-expressed. While developmentally, *Trpm8* is co-expressed in a subset of *Trpv1*-lineage neurons in the mouse (Mishra et al., 2011) pharmacologic ablation of Trpv1+ fibers in adult rodents suggests a further differentiation of a distinct subpopulation of *Trpm8*+/*Trpv1*- neurons (Cavanaugh et al., 2009) consistent with our anatomical findings. This is also consistent with ISH and single-cell RNA-Seq in DRG and trigeminal ganglion showing very low levels of *Trpv1* in *Trpm8*+ neurons (Kobayashi et al., 2005; Von Buchholtz et al., 2020). The idea of multiple *Trpm8*+ populations (Xing et al., 2006), where the *Trpv1*-coexpressing population may be more nociceptive in nature would potentially explain our results in context of the broader literature. We also cannot rule out an interaction between these input pathways at the level of the spinal cord, where Trpv1-specific and Trpm8-specific input pathways may be processed together in spinal circuits. For example, it is known that there is some interaction between Trpv1-specific and Trpm8-specific behavioral outcomes (Anderson et al., 2014).

The tetrodotoxin-resistant sodium channel subunit NaV1.9 (encoded by *Scn11a*) has come into focus as a potential nociceptor-specific target for the development of new non-opioid analgesics. Alongside two other sodium channel subunits in this family (*Scn9a* and *Scn10a*), *Scn11a* has been suggested as an interesting and understudied mediator of pain signaling. For example, the related ion channel subunit gene, *Scn9a* has been extensively studied in rodents and humans where it is strongly linked to pain and nociceptive signaling (Hisama et al., 1993; Dib-Hajj et al., 2005, 2007, 2013; Yang et al., 2018). Similarly, *Scn10a* has been used extensively as a nociceptive specific marker (Akopian et al., 1996; Dib-Hajj et al., 1999; Stirling et al., 2005; Shiers et al., 2020). The current study focuses on *Scn11a* largely due to the fact that it has been studied less intensively than *Scn9a* and *Scn10a*, and because mutations in this gene are associated with pain insensitivity or episodic pain syndromes in humans (Leipold et al., 2013; Zhang et al., 2013). For example, NaV1.9 has been found to be enriched in cells in the *Trpv1* lineage (Goswami et al., 2014) or depleted with TRPV1 agonist treatment (Isensee et al., 2014; Sapio et al., 2018), suggesting specificity for the nociceptive population. This channel has also been implicated in nociception and hyperalgesia in animal models, suggesting potential utility as an analgesic pharmacological target (Priest et al., 2005). NaV1.9 is required for cold-triggered nociception in the mouse (Lolignier et al., 2015), consistent with our observation that *Scn11a* is found in high-expressing *Trpm8*+ neurons. However, our staining shows a broad expression pattern of *Scn11a* consistent with involvement in other nociceptive functions. For example, *Scn11a* is frequently co-expressed with *Trpv1*, which is consistent with human mutation studies of NaV1.9 in nociception in which loss or gain of function *SCN11A* mutations can cause pain insensitivity or episodic pain syndromes (Leipold et al., 2013; Zhang et al., 2013). The phenotype depends on the location of mutation in the channel. Importantly, the finding that gain of function mutations that inhibit transmitter release leads to pain insensitivity suggests that NaV1.9 is expressed in enough nociceptive afferents to be sufficient to support a pain insensitive phenotype. These findings are also corroborated by other studies using single cell RNA-Seq showing that *Scn11a* is expressed in *Trpv1*+ cells, but also in several other classes of sensory afferents (Li et al., 2016; Sapio et al., 2018).

In our staining analysis, we examined secreted phosphoprotein 1 (*Spp1*) positive neurons, which are non-nociceptive large diameter primary afferents that innervate muscle spindles. The *Spp1* gene encodes osteopontin (Ichikawa et al., 2000). While the function of osteopontin in neurons has not been determined, it has been suggested as a regulator of myelination, and hypothesized that its expression is related to the maintenance or formation of axons with high conduction velocities (Higo et al., 2010). These neurons are thought to be chiefly implicated in proprioception (Ichikawa et al., 2000; Usoskin et al., 2015; Saito-Diaz et al., 2021). Anatomically, osteopontin immunoreactivity localizes to spiral axon terminals in muscle spindle fibers. In our staining, we found that *Spp1*+ cells were negative for *Trpv1* and *Oprm1*, but co-expressed *Oprl1*. These neurons were also generally large, and their staining pattern, showing distinction from *Trpv1* expressors, further suggests their non-nociceptive nature in the DRG.

The current study has several limitations that must be addressed in future studies. For the present investigation, experiments are conducted in male rats, and as such may not be fully reflective of the neuroanatomy and neurochemistry in the mouse or across both sexes (Sadler et al., 2022). For behavioral studies, the rat has many advantages compared to the mouse, however, at this point in time, a large set of molecular-genetic rat manipulations are not available for investigation as they are for the mouse nervous system (Ellenbroek and Youn, 2016; Homberg et al., 2017). Thus, the use of multiplex fluorescence represents a concerted effort to assemble the combinatorial molecular-cellular expression patterns in rat that mediate algesia and analgesia in sensory ganglia. The ultimate goal of these investigations is to gain insights into conserved molecular biological phenomena in both sexes and in humans. Future studies will concentrate on direct human investigation in male and female organ donor tissue to corroborate and extend these findings into human health, disease, and pharmacology (Iadarola et al., 2022), as such studies are more likely to yield information that may be more clinically relevant and applicable. As an additional area for future directions, much attention has been paid to the use of single cell and single nucleus RNA-Seq for the determination and characterization of neuronal cell types in the DRG in mouse, monkey and human (Usoskin et al., 2015; Kupari et al., 2021; Tavares-Ferreira et al., 2022). These studies are useful in the determination of cell types but often lack the precision to determine the expression profile of an individual gene. In the existing databases, the opioid receptors are weakly detected, and probably encountered a limit of detection. Additionally, for genes such as *Trpv1* and *Trpm8*, the mouse and monkey databases have shown correlation between *Trpv1* and *Trpm8*, where the cell population with the highest level of one also has the highest level of the other, which is in direct contrast to our findings (Usoskin et al., 2015; Kupari et al., 2021). However, this could be due to multiple technical issues or species differences and is beyond the scope of the current investigation. Finally, another limitation is that this study does not assess the impact of pain and nerve injury on expression and co-expression of these markers. Nerve injury in particular induces strong transcriptional changes and may alter the baseline expression patterns of nociceptive and analgesic targets (Ray et al., 2018; Sapio et al., 2020a). Future studies can expand upon these foundational data to understand pain conditions.

While the field of nociceptive neuroscience has expended substantial effort over many years toward understanding key receptors involved in nociceptive circuits, there is a need for exact delineation of neuronal populations that contribute to clinical pain and pain control as well as identification of molecular signatures which can define these distinct populations. Our study utilized multiplex high sensitivity mRNA *in situ* hybridization, which allowed us to obtain precise answers, leading to enhanced clarity of these issues. Importantly, our findings suggest that the medium to low expressing *Trpv1*+ neurons indeed represent the population that transmit nociceptive signaling associated with tissue-damage and this population is coincident with opioid-induced anti-nociception. Deeper analysis of the coexpression matrix of these nociceptive and analgesic target genes provides additional rationale for identifying susceptible neuronal populations for early-stage novel therapeutics development, with either pharmacological agents or other methods such as *in vivo* gene transfer, by better defining the neuronal populations directly responsible for clinically relevant pain and pain control.

## Data Availability Statement

The original contributions presented in the study are included in the article/Supplementary Material, further inquiries can be directed to the corresponding author.

## Ethics Statement

The animal study was reviewed and approved by NIH Clinical Center.

## Author Contributions

The project was conceptualized by MI, WM, and MS. Experiments were performed by WM, with technical involvement from MS, DM, and TG. Staining and slide scanning was performed by WM with assistance from AM and supervision from DM in fluorescence microscopy and image analysis. Stained sections were analyzed in Adobe Photoshop by WM with assistance from AM and MS. Visualizations, final figures, and formal analysis were generated primarily by MS, with assistance from WM, AM, and MD. Initial drafts of the manuscript were prepared by MS, WM, and MI with editing and suggestions from all of the authors. Funding was obtained by WM, DM, MI, and AM. The project was supervised by MS, AM, and MI. All authors revised and approved the final manuscript.

## Funding

Funding for this work was supported by the Intramural Research Program of the NIH Clinical Center (1ZIACL090033-09, 1ZIACL090034-09, and 1ZIACL090035-08 to AM), and the NINDS. This work was also supported by a funds from the National Center for Complementary and Integrative Health (1ZIAAT000017-03), and the Office of Behavioral and Social Sciences Research. TG was supported by the Japan Society for the Promotion of Science Overseas Research Fellowship. This research was made possible through the National Institutes of Health (NIH) Medical Research Scholars Program, a public-private partnership supported jointly by the NIH and generous contributions to the Foundation for the NIH from the Doris Duke Charitable Foundation, Genentech, the American Association for Dental Research, the Colgate-Palmolive Company, Elsevier, alumni of student research programs, and other individual supporters *via* contributions to the Foundation for the National Institutes of Health.

## Conflict of Interest

The authors declare that the research was conducted in the absence of any commercial or financial relationships that could be construed as a potential conflict of interest.

## Publisher's Note

All claims expressed in this article are solely those of the authors and do not necessarily represent those of their affiliated organizations, or those of the publisher, the editors and the reviewers. Any product that may be evaluated in this article, or claim that may be made by its manufacturer, is not guaranteed or endorsed by the publisher.
