# Supplementary material for: Anatomical Analysis of Transient Potential Vanilloid Receptor 1 (Trpv1+) and Mu-Opioid Receptor (Oprm1+) Co-expression in Rat Dorsal Root Ganglion Neurons
Source: Front Mol Neurosci. 2022 Jul 7;15:926596. doi: 10.3389/fnmol.2022.926596 (PMC9302591; doi:10.3389/fnmol.2022.926596)
Supplement: Supplementary file 1 [file Data_Sheet_1.docx]

Supplementary Material

*Anatomical analysis of Transient potential vanilloid receptor 1 (*Trpv1*+) and Mu-opioid receptor (*Oprm1*+) coexpression in rat dorsal root ganglion neurons*

## Supplementary Figures

| **Fluorophore** | **Exciter** | **Dichroic** | **Emitter** |
| --- | --- | --- | --- |
| DAPI | FF01-340/26 | FF458-Di02 | FF01-482/25 |
| Alexa Fluor 488, Opal 520 | FF01-494/20 | FF506-Di03 | FF01-527/20 |
| Alexa Fluor 546, Opal 570 | FF01-535/22 | FF560-Di01 | FF01-580/23 |
| Alexa Fluor 594, Opal 620 | FF01-586/20 | FF605-Di02 | FF01-628/32 |
| Alexa Fluor 680, Opal 690 | FF01-680/22 | FF705-Di01 | FF01-720/13 |

**Supplementary Table 1. Exact specifications of custom filter sets used in imagine experiments.** These specifications are described in detail in (Maric et al., 2021).

**
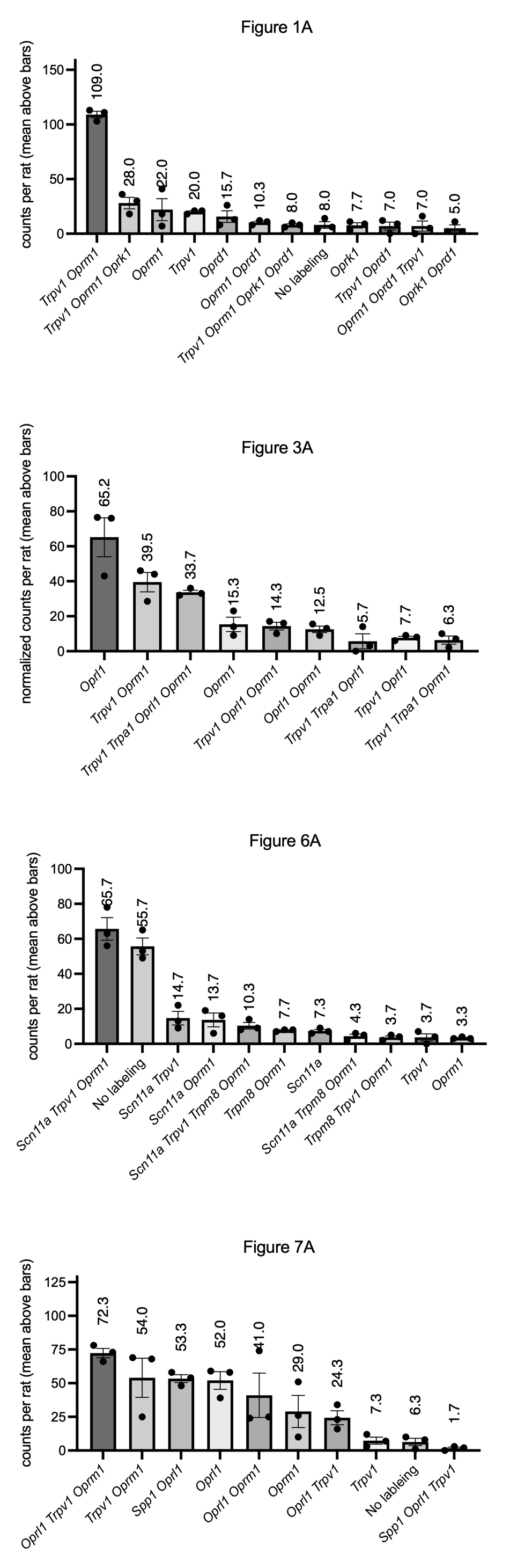
Supplementary Figure 1. Individual values for N=3 male rats in coincidence plots.** For plots made in UpSet (Figure 1A, 3A, 6A and 7A) aggregated values are shown as larger numbers of counts resulted in more consistent quantification of small subpopulations of neurons. Individual values were checked for consistency before pooling the N=3 male rats. The values for the pooled individuals are plotted. Numbers above each bar show the mean counts per animal. Note that in one animal in Figure 3A, there was uneven numbers of counts necessitating normalization to account for unequal cell counts in one animal. Error bars represent the standard error of the mean.


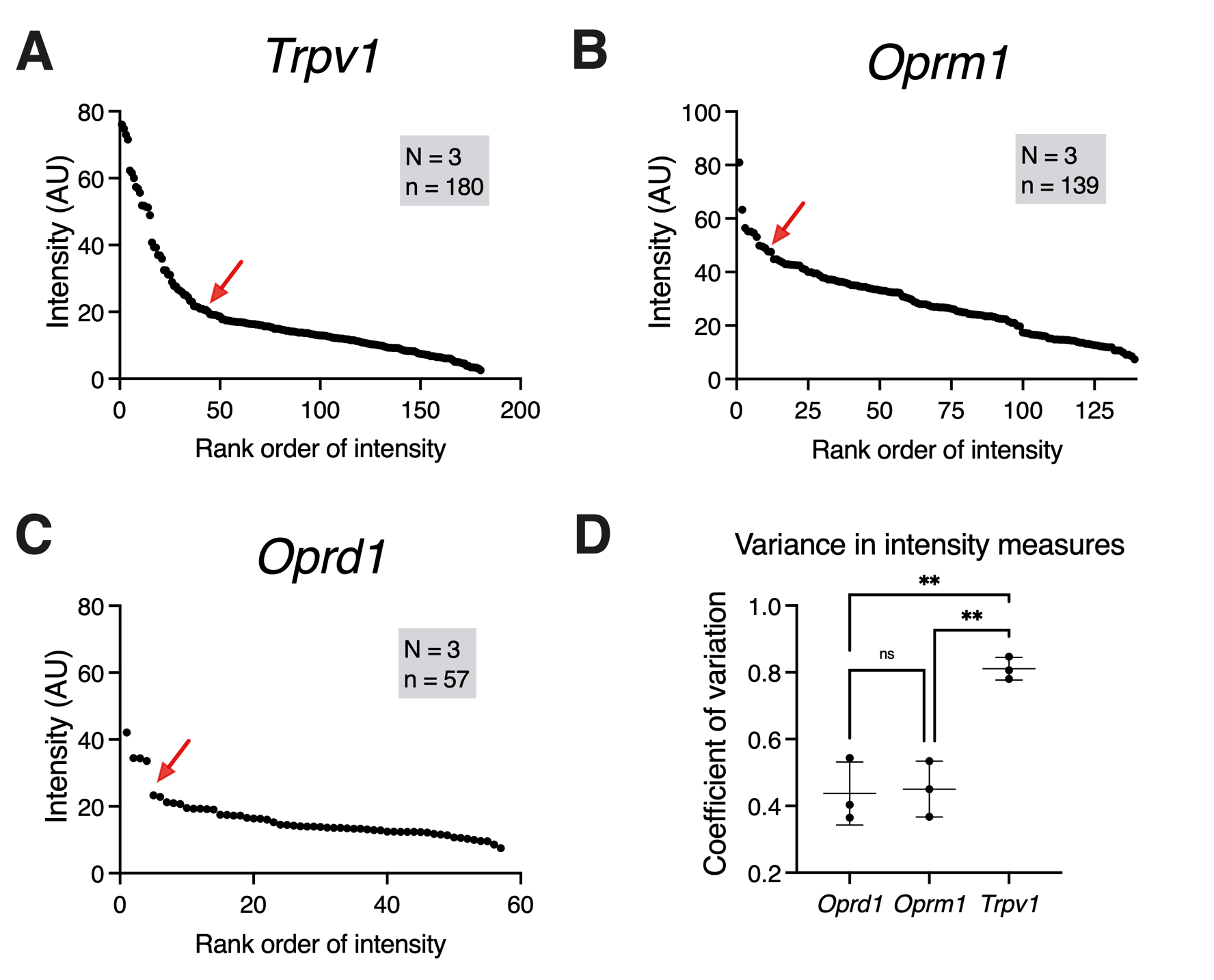


**Supplementary Figure 2. Analysis of intensity distribution and coefficient of variance of intensity for *Trpv1*, *Oprm1* and *Oprd1*.** We observed that *Trpv1* appeared to have very bright cells indicating quantitative differences in expression level corresponding to high and low expressors. To examine this phenomenon in relation to Oprm1 and Oprd1, we plotted the rank order of intensity from regions of interest drawn around cells in N=3 male rats. **A.** For *Trpv1* (n = 180 cells total), this plot shows a non-linearity marked by a subpopulation of *Trpv1* neurons with much higher expression. **B.** For *Oprm1*, the distribution appeared more linear with an inflection point at the leftmost part of the axis suggesting a small population with brighter *Oprm1* signal. **C.** For *Opd1*, the inflection point was also left-shifted indicating that only a small subset of neurons had distinctly bright expression. **D.** This was tested by measuring coefficient of variation for each of the three animals, identifying a significantly higher coefficient of variation for *Trpv1* signal among *Trpv1*+ cells (One-way ANOVA followed by Holm-Šídák's multiple comparisons test, Prism 9, GraphPad; **, *p* < 0.01; ns, not significant). As a technical note, the Trpv1 data in Panel A were performed separately from those in Figure 2F in order to capture quantitative data from the same animals/sections to directly compare these three signals as closely as possible. The average coefficient of variation for the data in Figure 2F was ~1.12, consistent with the idea that *Trpv1* intensity values are unevenly distributed.

**
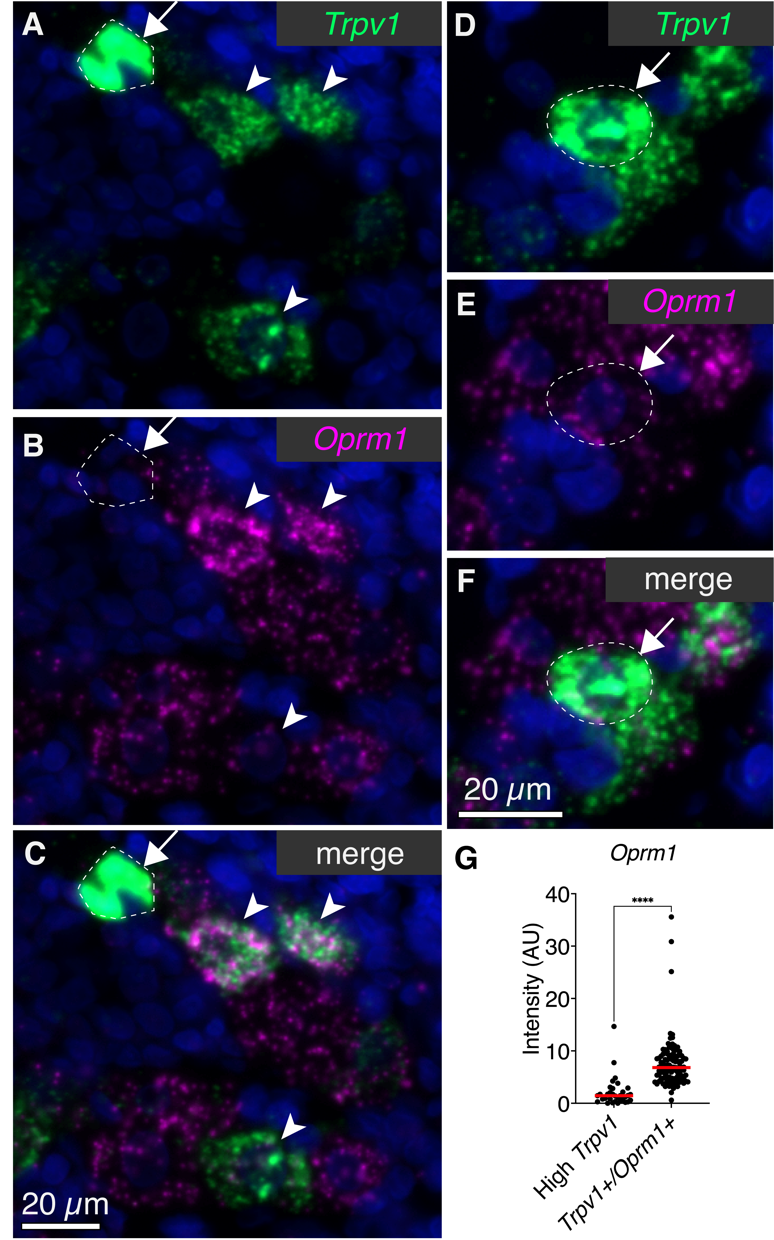
**

**Supplementary Figure 3. Analysis of *Oprm1* expression level in high Trpv1-expressing DRG neurons.** Based on the analysis in Figure 4F, we observed that most *Trpv1* neurons also express *Oprm1*, but that high *Trpv1* neurons are on average unlikely to express *Oprm1*+. **A.** A representative field is shown with a single high-*Trpv1* neuron (arrow, surrounded by dashed line), and three other *Trpv1*+ neurons (arrowheads). **B.** The same field is shown for *Oprm1*. Note that *Oprm1* is expressed in all three neurons marked by the arrowhead, but absent from the high *Trpv1* neuron. **C.** In the merged image, the co-expression of *Trpv1* and *Oprm1* is evident in the top two neurons marked by arrowheads, but is also present in the third neuron (although *Oprm1* expression is lower and obscured by the *Trpv1* signal.) **D.** Another field is shown for a high-*Trpv1* neuron expressing *Oprm1*. **E.** In general, we observed that high *Trpv1* neurons usually have low levels of *Oprm1* when *Orpm1+*. This can be seen in the *Oprm1* channel which shows low to moderate staining within the perikaryal (marked with dashed line). **F.** Merged image of *Trpv1* and *Oprm1* channels. **G.** Quantification was performed in Fiji by measuring areas of 8-bit grayscale image captures around the perikarya of neurons from several representative windows of DRG sections (N=3 rats). High *Trpv1* neurons on average had significantly less *Oprm1* than the average *Trpv1*+/*Oprm1*+ neuron (exclusive of high-*Trpv1* neurons). Significance testing was performed in Prism Graphpad 8 using a Mann-Whitney test; *p* < 0.0001; n = 33 cells high-*Trpv1* vs. n = 133 cells *Trpv1*+/*Oprm1*+; median 1.4 vs. median 6.8 in arbitrary units. Slides in the same comparison were scanned at the same time with identical parameters.


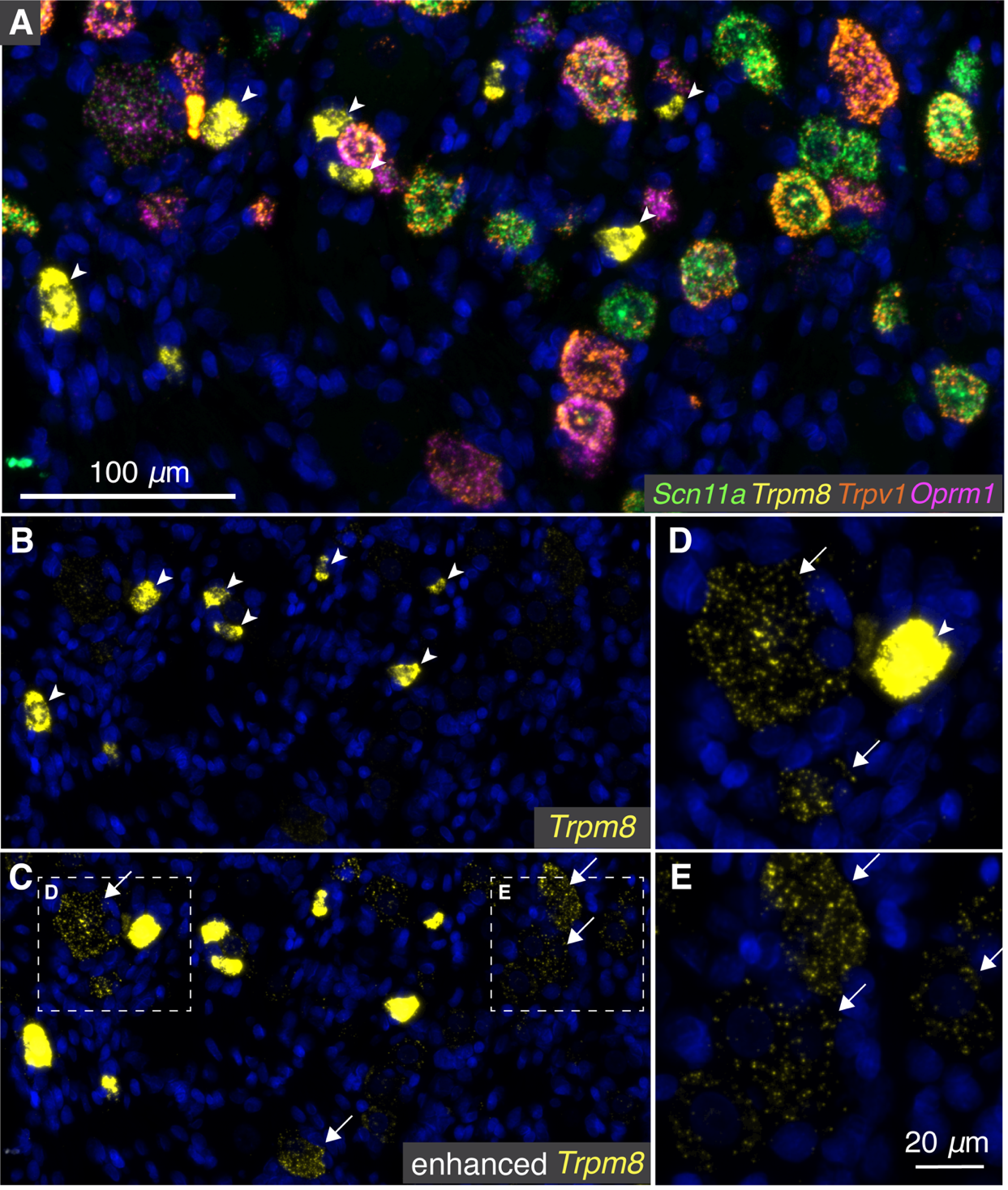


**Supplementary Figure 4. Validation of *Trpm8* low expressing neurons using an alternative probe.** Very low expressing *Trpm8*+ neurons were observed in images after enhancement (Figure 5). To validate that this result was due to specific staining, the *Trpm8* probe was redesigned to exclude the possibility of low affinity off target binding/amplification. **A.** Note the highly *Trpm8*+ neurons in the 4-plex stain (arrowheads). These small neurons appear brightly labeled for *Trpm8* (yellow). **B.** The Trpm8 channel is shown alone for better visibility. These neurons appear as bright yellow bodies. **C.** After enhancement of the brightness, lower intensity *Trpm8* neurons become visible similar to the stain shown in Figure 5 (indicated by arrows). Note that at this intensity the brightly *Trpm8*+ neurons are saturated. **D.** An enlargement of the enhanced image is shown with both a saturated *Trpm8*+ neuron (arrowhead) as well as two low expressing *Trpm8*+ neurons (arrows). **E.** A second field of low Trpm8+ neurons is shown.

**References**

MARIC, D., JAHANIPOUR, J., LI, X. R., SINGH, A., MOBINY, A., VAN NGUYEN, H., SEDLOCK, A., GRAMA, K. & ROYSAM, B. 2021. Whole-brain tissue mapping toolkit using large-scale highly multiplexed immunofluorescence imaging and deep neural networks. *Nat Commun,* 12**,** 1550.
